# Supplementary material for: Heterogeneous Photocatalysis as a Potent Tool for Organic Synthesis: Cross-Dehydrogenative C–C Coupling of N-Heterocycles with Ethers Employing TiO2/N-Hydroxyphthalimide System under Visible Light
Source: Molecules. 2023 Jan 17;28(3):934. doi: 10.3390/molecules28030934 (PMC9920906; doi:10.3390/molecules28030934)
Supplement: Supplementary file 1 [file molecules-28-00934-s001.zip › molecules-2152548-supplementary.pdf]

Supporting Information

Heterogeneous photocatalysis as a potent tool for organic synthesis: cross-dehydrogenative C–C coupling of *N*-heterocycles with ethers employing TiO_2/*N*-hydroxyphthalimide system under visible light

Elena R. Lopat'eva, Igor B. Krylov*, Oleg O. Segida, Valentina M. Merkulova, Alexey I. Ilovaisky and Alexander O. Terent'ev*

N. D. Zelinsky Institute of Organic Chemistry, Russian Academy of Sciences, 47 Leninsky Prospekt, Moscow 119991, Russia

* Correspondence: krylovigor@yandex.ru (I.B.K.); alterex@yandex.ru (A.O.T.)

Table of contents

General 3

The comparison of the developed method with the literature procedure 4

The determination of the side products of the studied process 5

Spectral data of the Minisci reaction products 11

General

In all experiments RT stands for 23-25 °C. ^{1}H and ^{13}C NMR spectra were recorded on a Bruker AVANCE II 300 and Bruker Fourier 300HD (300.13 and 75.47 MHz, respectively) spectrometers in $CDCl_3$ and DMSO-D_6. Residual signals of $CDCl_3$ (7.26 in ^{1}H NMR, 77.16 in ^{13}C NMR) were used as reference signals for precise chemical shift determination. FT-IR spectra were recorded on Bruker Alpha instrument. IR spectra were registered in KBr pellets for solid compounds, and liquid compounds were placed between two KBr windows to make a thin layer. High resolution mass spectra (HR-MS) were measured on a Bruker maXis instrument using electrospray ionization (ESI). The measurements were performed in a positive ion mode (interface capillary voltage – 4500 V); mass range from m/z 50 to m/z 3000 Da; external calibration with Electrospray Calibrant Solution (Fluka). A syringe injection was used for all acetonitrile solutions (flow rate 3 μL/min). Nitrogen was applied as a dry gas; interface temperature was set at 180 °C.

The comparison of the developed method with the literature procedure

The coupling products of the of THF with pyrazines (**3ga**, **3ha**) have not been previously described. We decided to test other known effective procedure [1] to compare the results with the results achieved by our method. The chosen literature method [1] works reliably for the coupling of 4-methylquinoline **1a** with dioxane **2d** (I, conditions A), while our system gives lower yields of product **3ad** (I, conditions B). When switching to pyrazine **1g** as a substrate we do not observe the desired product **3ga** using the literature procedure (II, conditions A). However, it was possible to obtain **3ga** using our photochemical approach (II, conditions B).

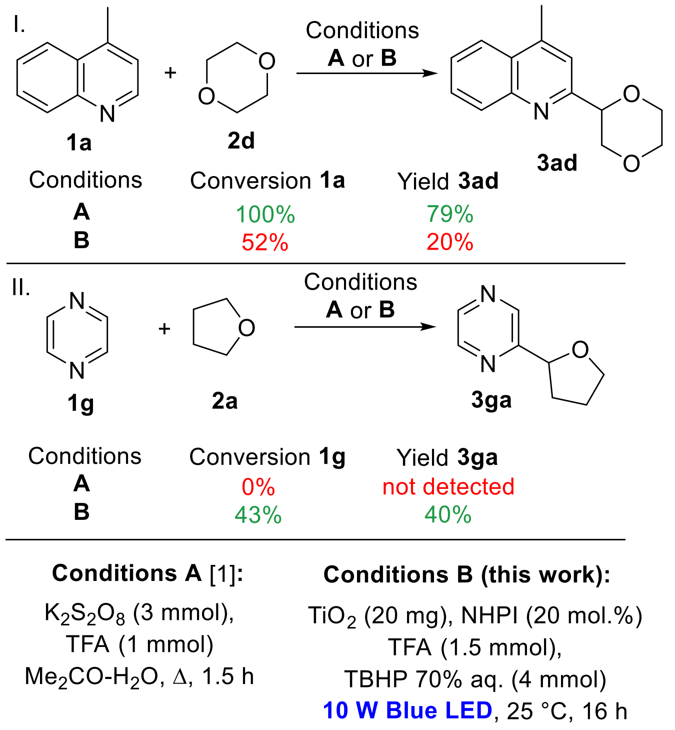

Experimental details for conditions A [1]. To a solution of heterocycle (1 mmol) in a mixture of acetone/H_2O (7 mL/3 mL) was added the corresponding ether (20 mmol, 20 equiv.), $K_2S_2O_8$ (811 mg, 3 mmol, 3 equiv.) and TFA (80μL, 1 mmol). The mixture was then stirred for 1.5h under reflux. The reaction was quenched with saturated aqueous $NaHCO_3$ (16 mL) and extracted with ethyl acetate (2 × 25 mL). The combined organic phases were dried over $MgSO_4$ and concentrated under reduced pressure. The crude product was further purified by chromatography on silica gel (Petroleum ether/EtOAc = 2/1) to give the desired product.

For experimental details of conditions **B**, see the Experimental details for the Schemes 2 and 3.

The determination of the side products of the studied process

To identify the oxidation products of **3aa**, the ^{1}H, ^{13}C NMR and HRMS spectra were recorded for the reaction mixture in conditions for Scheme 4, A (See 3. Materials and Methods, Experimental details for Scheme 4, A). The peroxide 2-(2-hydroperoxytetrahydrofuran-2-yl)-4-methylquinoline **3aa'** was recognized as the major product. The characteristic ^{13}C signal corresponding to the **C**-OOH of hydroperoxide is 113.3, which is in a good agreement with the literature data for α-alkoxyhydroperoxides [2]. HR-MS spectrum confirmed the formation of **3aa'**.

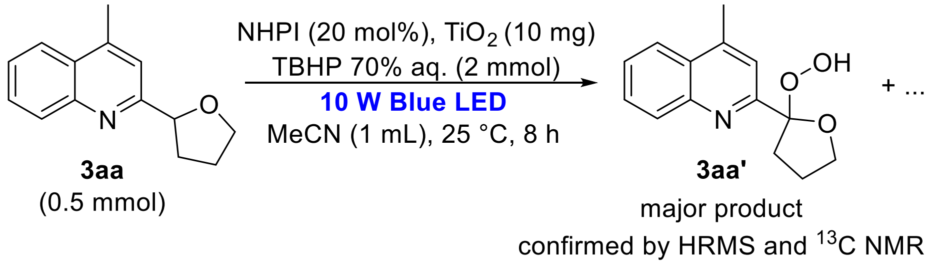

2-(2-hydroperoxytetrahydrofuran-2-yl)-4-methylquinoline 3aa'. $^{13}C\{^{1}H\}$NMR (75.48 MHz, $CDCl_3$) δ 159.4, 146.0, 145.7, 128.8, 128.7, 127.2, 126.1, 123.4, 119.7, 113.3, 69.5, 36.8, 24.8, 19.0. HR-MS (ESI): m/z = 246.1125, calcd. for $C_{14}H_{15}NO_3+H^+$: 246.1123.

1. McCallum, T.; Jouanno, L.-A.; Cannillo, A.; Barriault, L. Persulfate-Enabled Direct C–H Alkylation of Heteroarenes with Unactivated Ethers. *Synlett* **2016**, *27*, 1282–1286, doi:10.1055/s-0035-1561338.
2. Terent'ev, A.O.; Platonov, M.M.; Krylov, I.B.; Chernyshev, V.V.; Nikishin, G.I. Synthesis of 1-Hydroperoxy-1′-Alkoxyperoxides by the Iodine-Catalyzed Reactions of Geminal Bishydroperoxides with Acetals or Enol Ethers. *Org. Biomol. Chem.* **2008**, *6*, 4435, doi:10.1039/b809661a.

	Parameter	Value
1	Title	EL946.{1H}.1.fid
2	Solvent	CDCl3
3	Temperature	298.0
4	Pulse Sequence	zg
5	Number of Scans	1
6	Receiver Gain	32.0
7	Relaxation Delay	0.1000
8	Pulse Width	14.7000
9	Acquisition Time	2.7724
10	Spectrometer Frequency	300.23
11	Spectral Width	5882.4
12	Lowest Frequency	-1087.3
13	Nucleus	1H
14	Acquired Size	16308
15	Spectral Size	65536

^{1}H NMR (300.13 MHz, $CDCl_3$)

2-(2-hydroperoxytetrahydrofuran-2-yl)-4-methylquinoline 3aa’ in mixture

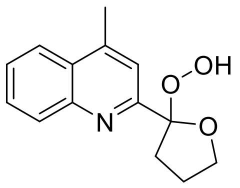

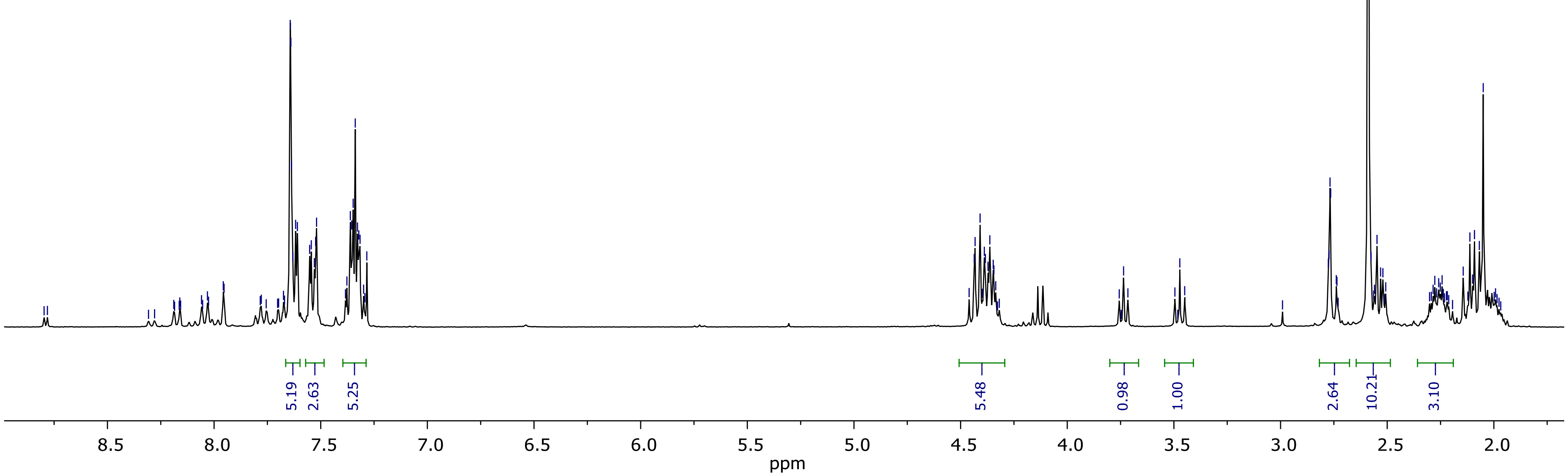

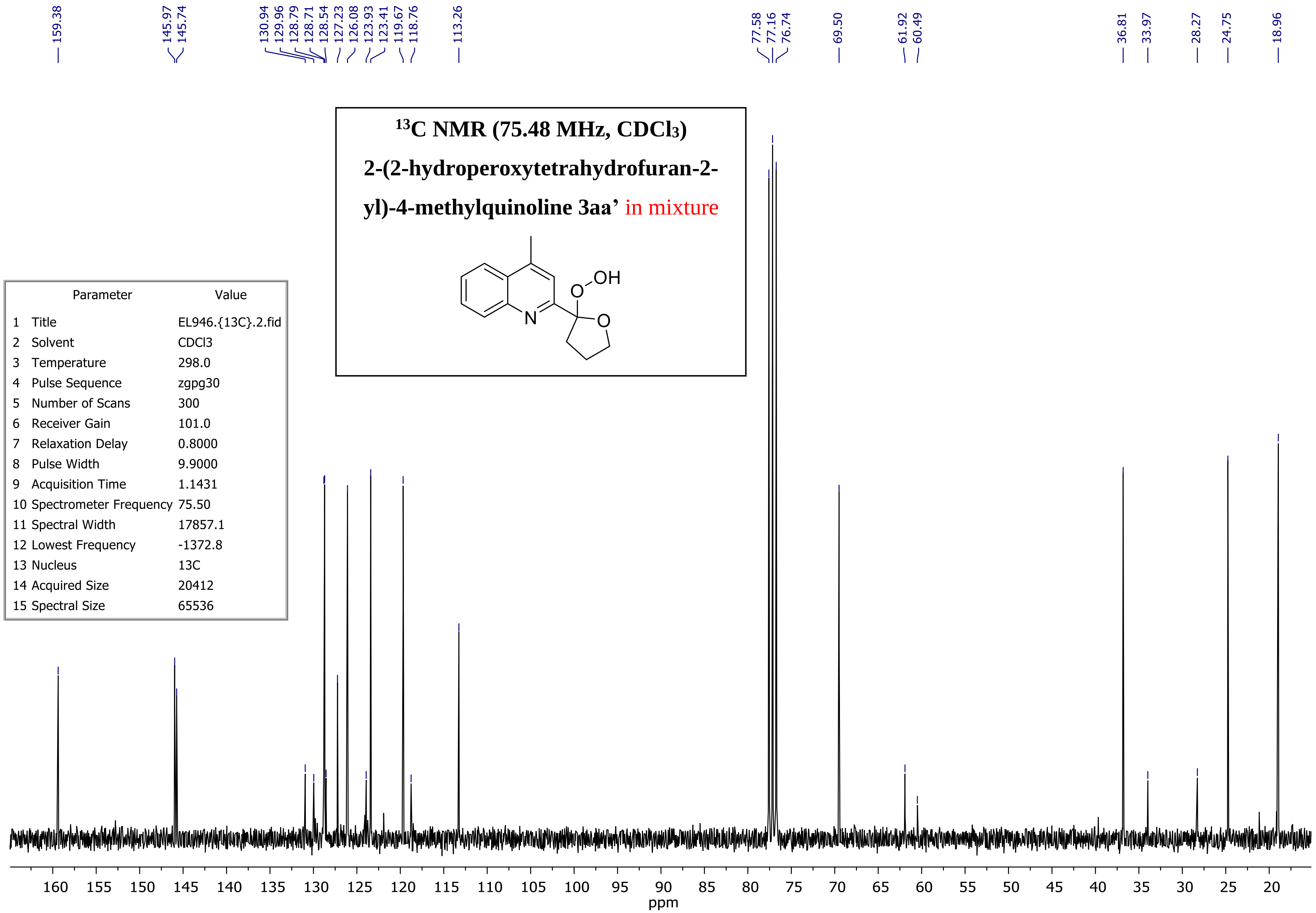

159.38
145.97
145.74
130.94
129.96
128.79
128.71
128.54
127.23
126.08
123.93
123.41
119.67
118.76
113.26
77.58
77.16
76.74
69.50
61.92
60.49
36.81
33.97
28.27
24.75
18.96
^{13}C NMR (75.48 MHz, CDCl$_3$)
2-(2-hydroperoxytetrahydrofuran-2-yl)-4-methylquinoline 3aa’ in mixture
OH
O
N
O
Parameter | Value
1 Title | EL946.{13C}.2.fid
2 Solvent | CDCl3
3 Temperature | 298.0
4 Pulse Sequence | zgpg30
5 Number of Scans | 300
6 Receiver Gain | 101.0
7 Relaxation Delay | 0.8000
8 Pulse Width | 9.9000
9 Acquisition Time | 1.1431
10 Spectrometer Frequency | 75.50
11 Spectral Width | 17857.1
12 Lowest Frequency | -1372.8
13 Nucleus | 13C
14 Acquired Size | 20412
15 Spectral Size | 65536
160 155 150 145 140 135 130 125 120 115 110 105 100 95 90 85 80 75 70 65 60 55 50 45 40 35 30 25 20
ppm

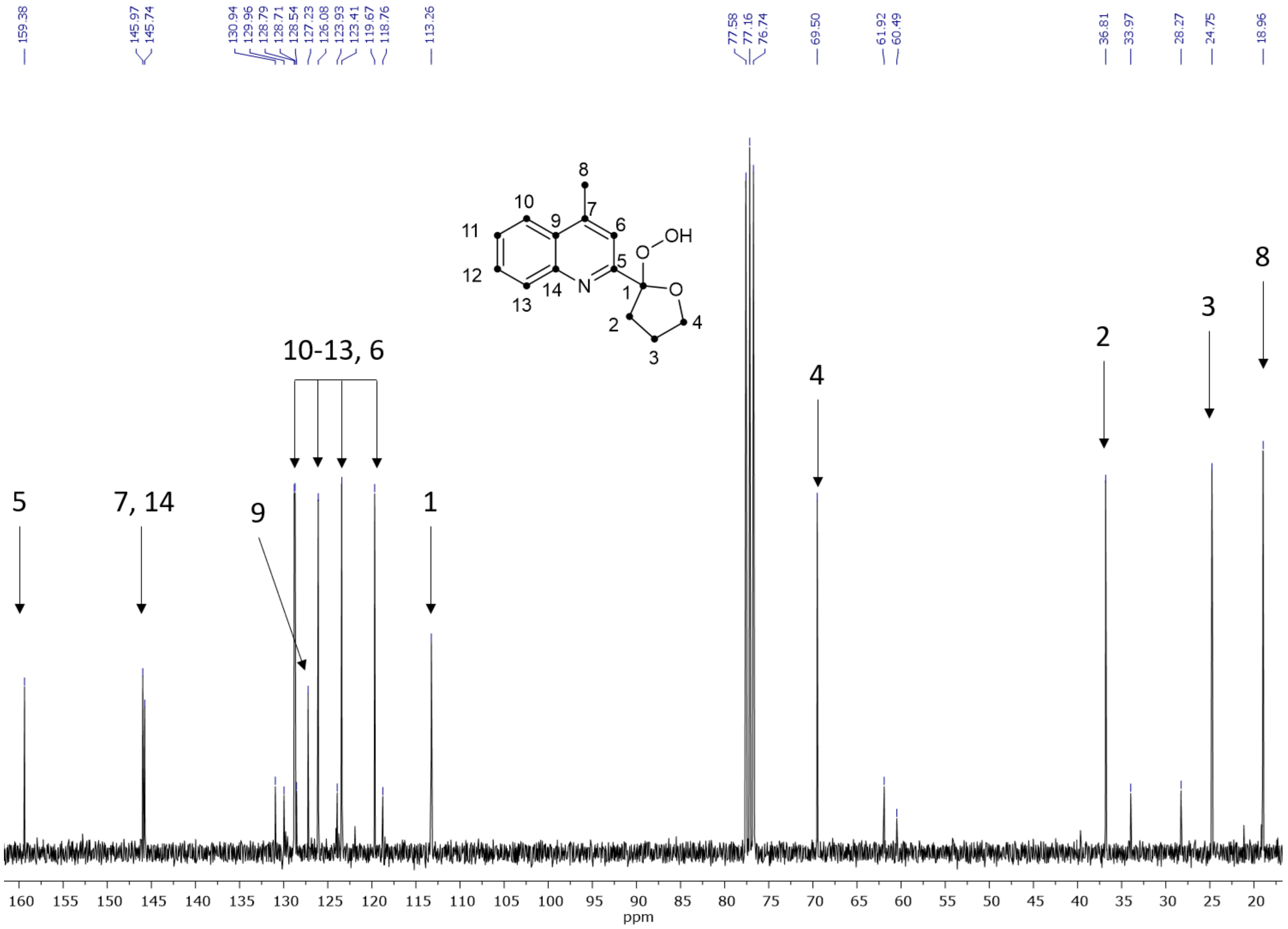
159.38
145.97
145.74
130.94
129.96
128.79
128.71
128.54
127.23
126.08
123.93
123.41
119.67
118.76
113.26
77.58
77.16
76.74
69.50
61.92
60.49
36.81
33.97
28.27
24.75
18.96
OH
O
N
5
7, 14
9
10-13, 6
1
4
2
3
8
ppm
160
155
150
145
140
135
130
125
120
115
110
105
100
95
90
85
80
75
70
65
60
55
50
45
40
35
30
25
20

Display Report

Analysis Info

Analysis Name	D:\Data\Chizhov\Terentiev\Lopat'eva\ll-1611-a_&clblow.d	Acquisition Date	14.10.2022 16:08:12
Method	tune_low.m	Operator	BDAL@DE
Sample Name	/TERN LL-1611-A	Instrument / Ser#	micrOTOF 10248
Comment	CH3CN 100 %, dil. >2000, calibrant added		

Acquisition Parameter

Source Type	ESI	Ion Polarity	Positive	Set Nebulizer	0.4 Bar
Focus	Not active			Set Dry Heater	180 °C
Scan Begin	50 m/z	Set Capillary	4500 V	Set Dry Gas	4.0 l/min
Scan End	3000 m/z	Set End Plate Offset	-500 V	Set Divert Valve	Waste

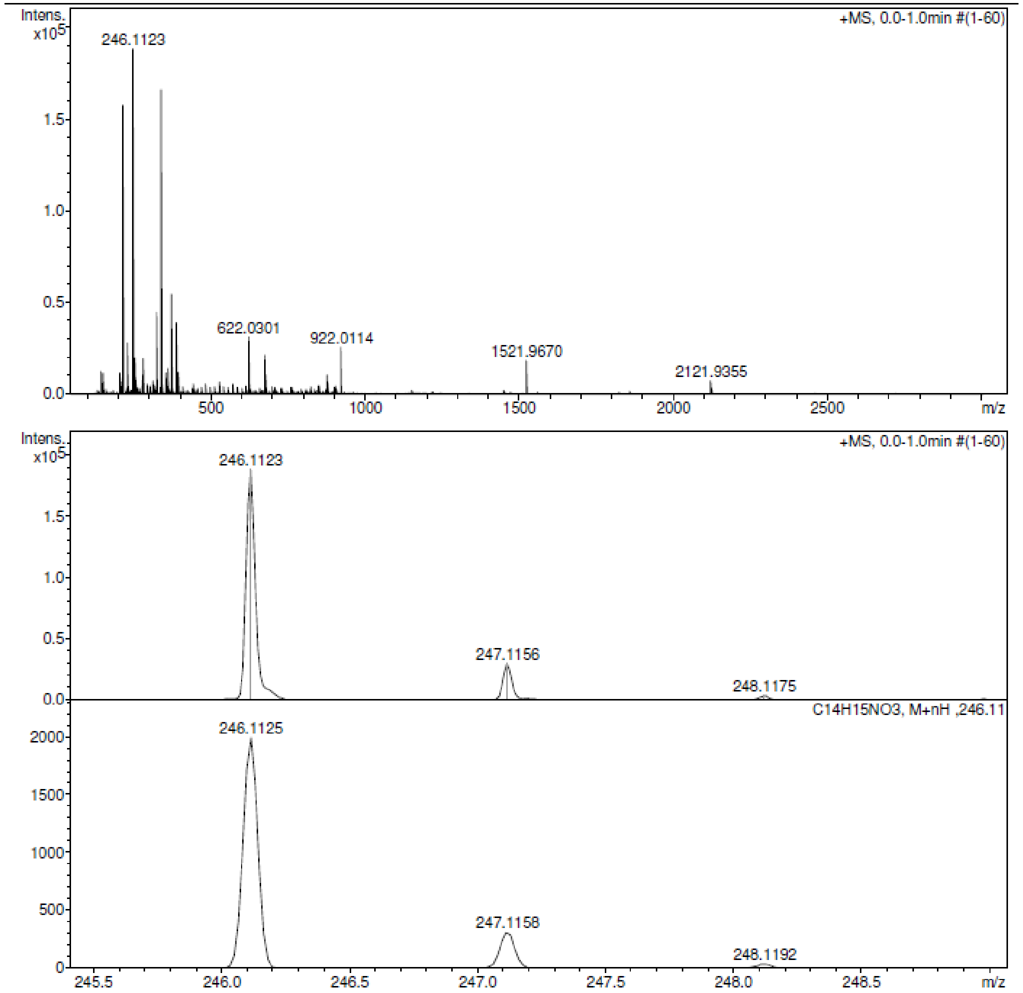

Spectral data of the Minisci reaction products

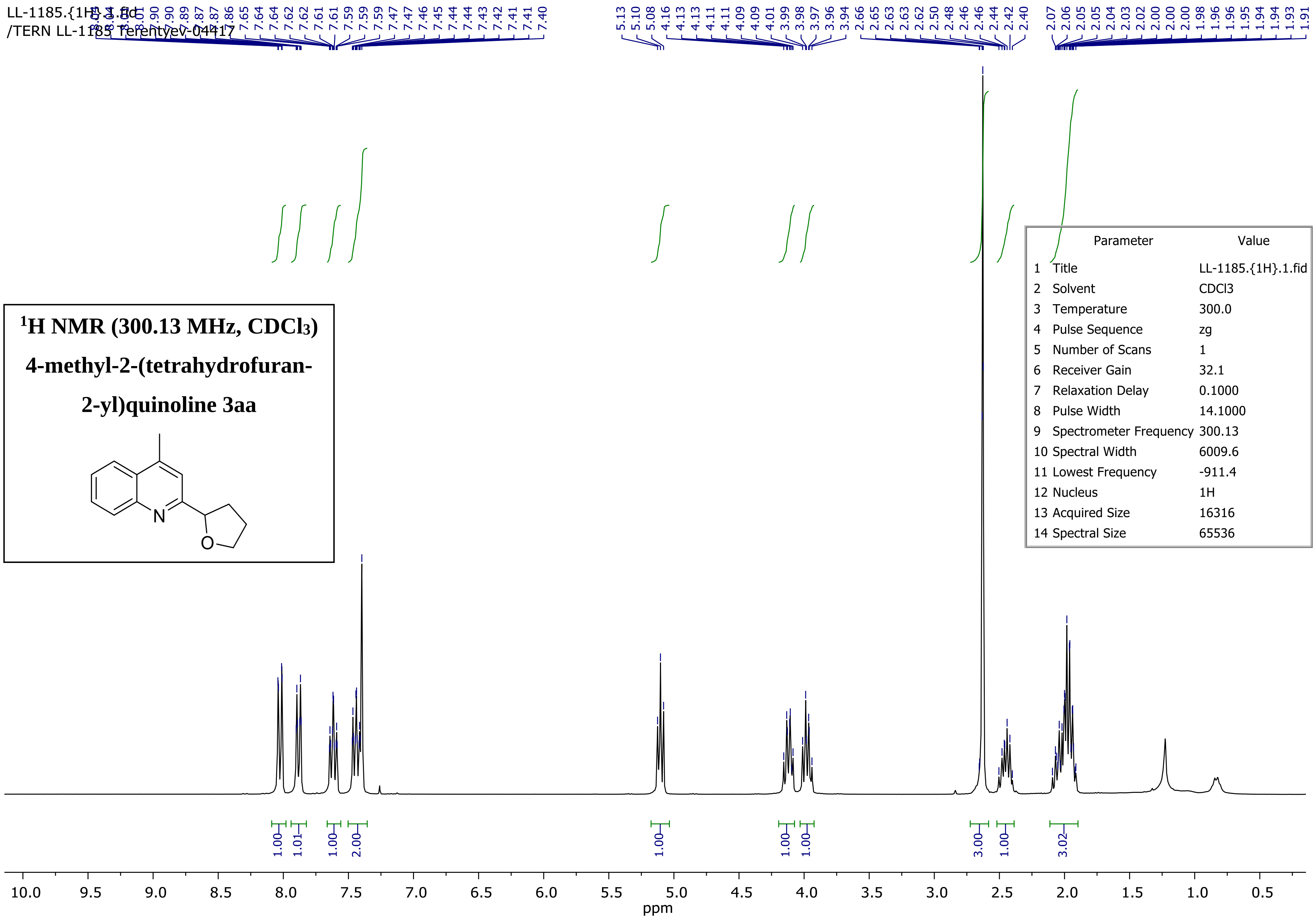

^{1}H NMR (300.13 MHz, $CDCl_3$)

4-methyl-2-(tetrahydrofuran-2-yl)quinoline 3aa

	Parameter	Value
1	Title	LL-1185.{1H}.1.fid
2	Solvent	CDCl3
3	Temperature	300.0
4	Pulse Sequence	zg
5	Number of Scans	1
6	Receiver Gain	32.1
7	Relaxation Delay	0.1000
8	Pulse Width	14.1000
9	Spectrometer Frequency	300.13
10	Spectral Width	6009.6
11	Lowest Frequency	-911.4
12	Nucleus	1H
13	Acquired Size	16316
14	Spectral Size	65536

LL-1185.{13C}.2.fid
/TERN LL-1185 Terentyev-04417

^{13}C NMR (75.48 MHz, $CDCl_3$)

4-methyl-2-(tetrahydrofuran-2-yl)quinoline 3aa

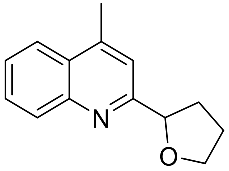

	Parameter	Value
1	Title	LL-1185.{13C}.2.fid
2	Solvent	CDCl3
3	Temperature	300.0
4	Pulse Sequence	zgpg30
5	Number of Scans	80
6	Receiver Gain	214.2
7	Relaxation Delay	0.8000
8	Pulse Width	9.3000
9	Spectrometer Frequency	75.48
10	Spectral Width	18028.8
11	Lowest Frequency	-1472.6
12	Nucleus	13C
13	Acquired Size	16316
14	Spectral Size	65536

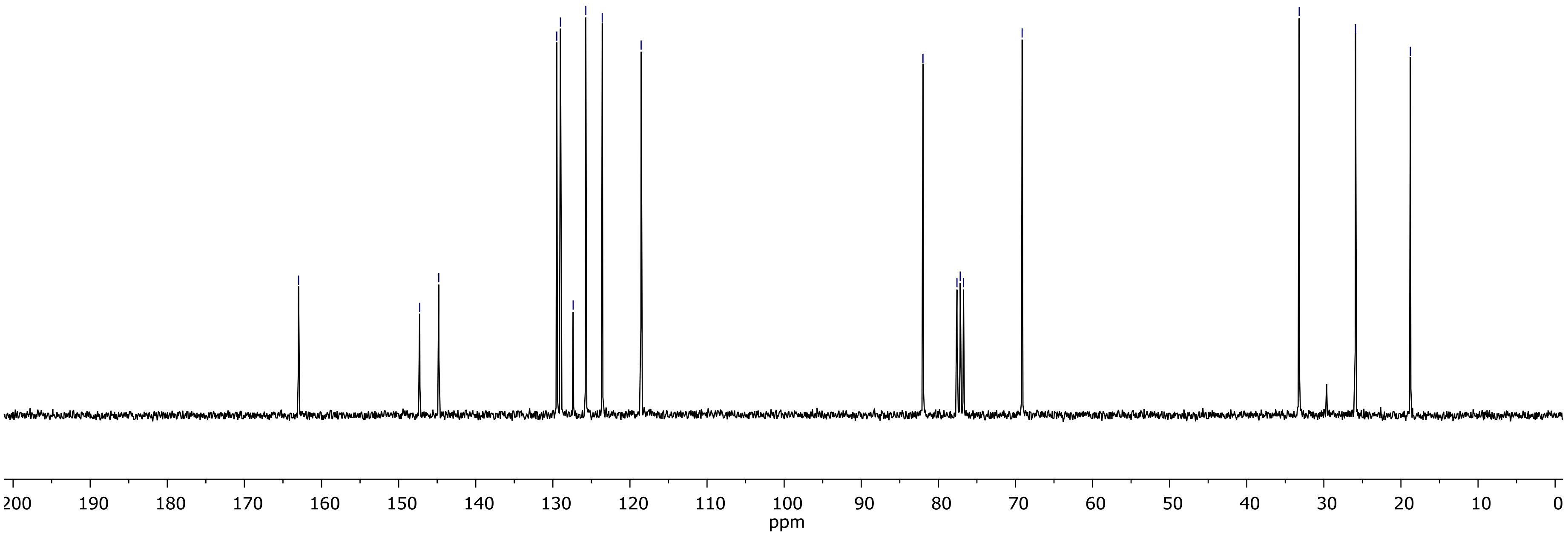

^{1}H NMR (300.13 MHz, $CDCl_3$)

4-methyl-2-(5-methyltetrahydrofuran-2-yl)quinoline 3ab

	Parameter	Value
1	Title	EL1000.{1H}.1.fid
2	Solvent	CDCl3
3	Temperature	298.0
4	Pulse Sequence	zg
5	Number of Scans	1
6	Receiver Gain	32.0
7	Relaxation Delay	0.1000
8	Pulse Width	14.7000
9	Acquisition Time	2.7724
10	Spectrometer Frequency	300.23
11	Spectral Width	5882.4
12	Lowest Frequency	-1094.5
13	Nucleus	1H
14	Acquired Size	16308
15	Spectral Size	65536

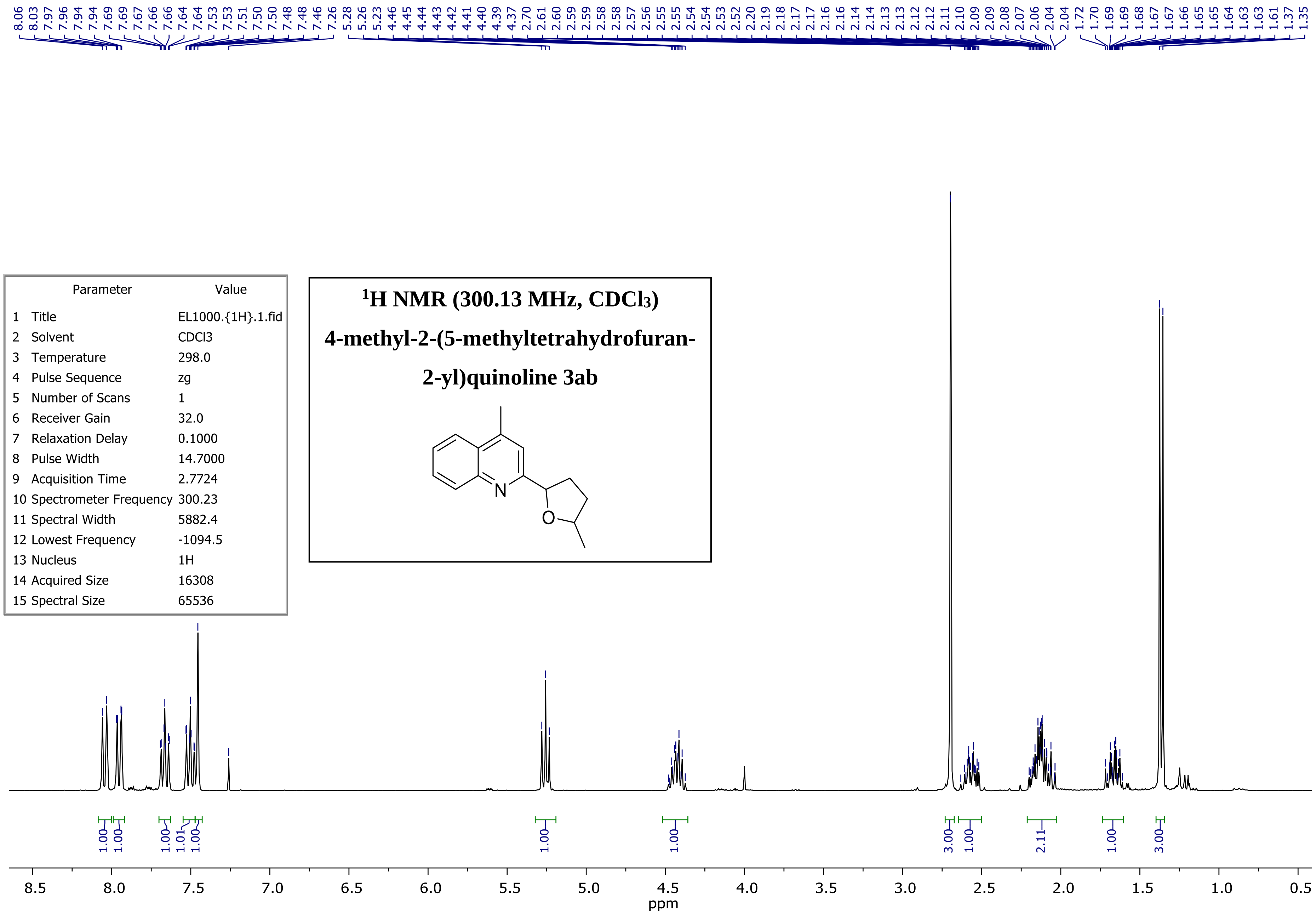

^{13}C NMR (75.48 MHz, $CDCl_3$)

4-methyl-2-(5-methyltetrahydrofuran-2-yl)quinoline 3ab

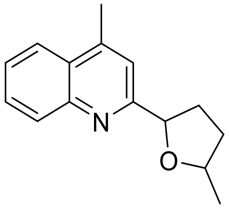

	Parameter	Value
1	Title	EL1000.{13C}.2.fid
2	Solvent	CDCl3
3	Temperature	298.0
4	Pulse Sequence	zgpg30
5	Number of Scans	300
6	Receiver Gain	101.0
7	Relaxation Delay	0.8000
8	Pulse Width	9.9000
9	Acquisition Time	1.1431
10	Spectrometer Frequency	75.50
11	Spectral Width	17857.1
12	Lowest Frequency	-1372.9
13	Nucleus	13C
14	Acquired Size	20412
15	Spectral Size	65536

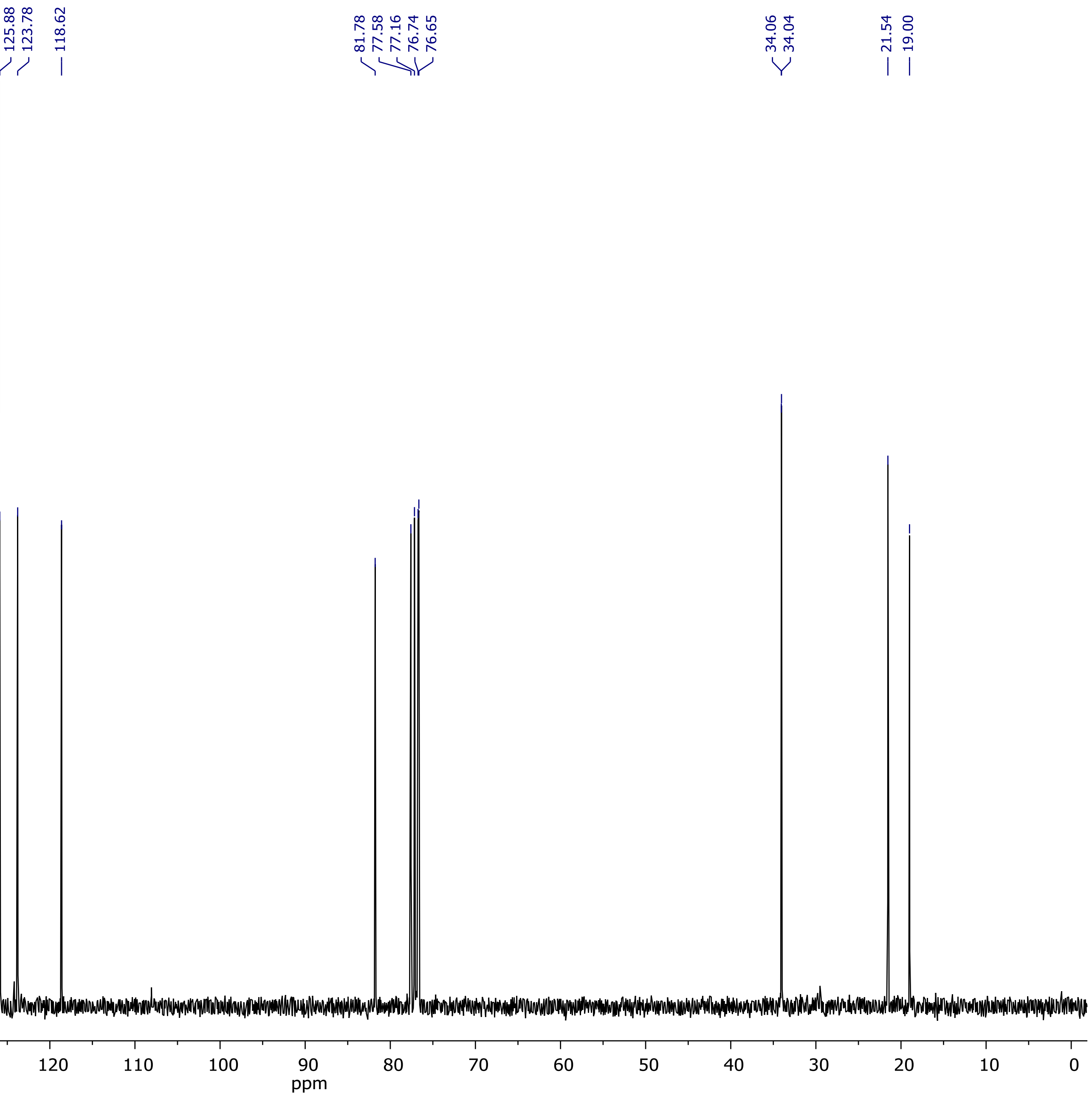

^{1}H-^{13}C HMBC

4-methyl-2-(5-methyltetrahydrofuran-2-yl)quinoline 3ab

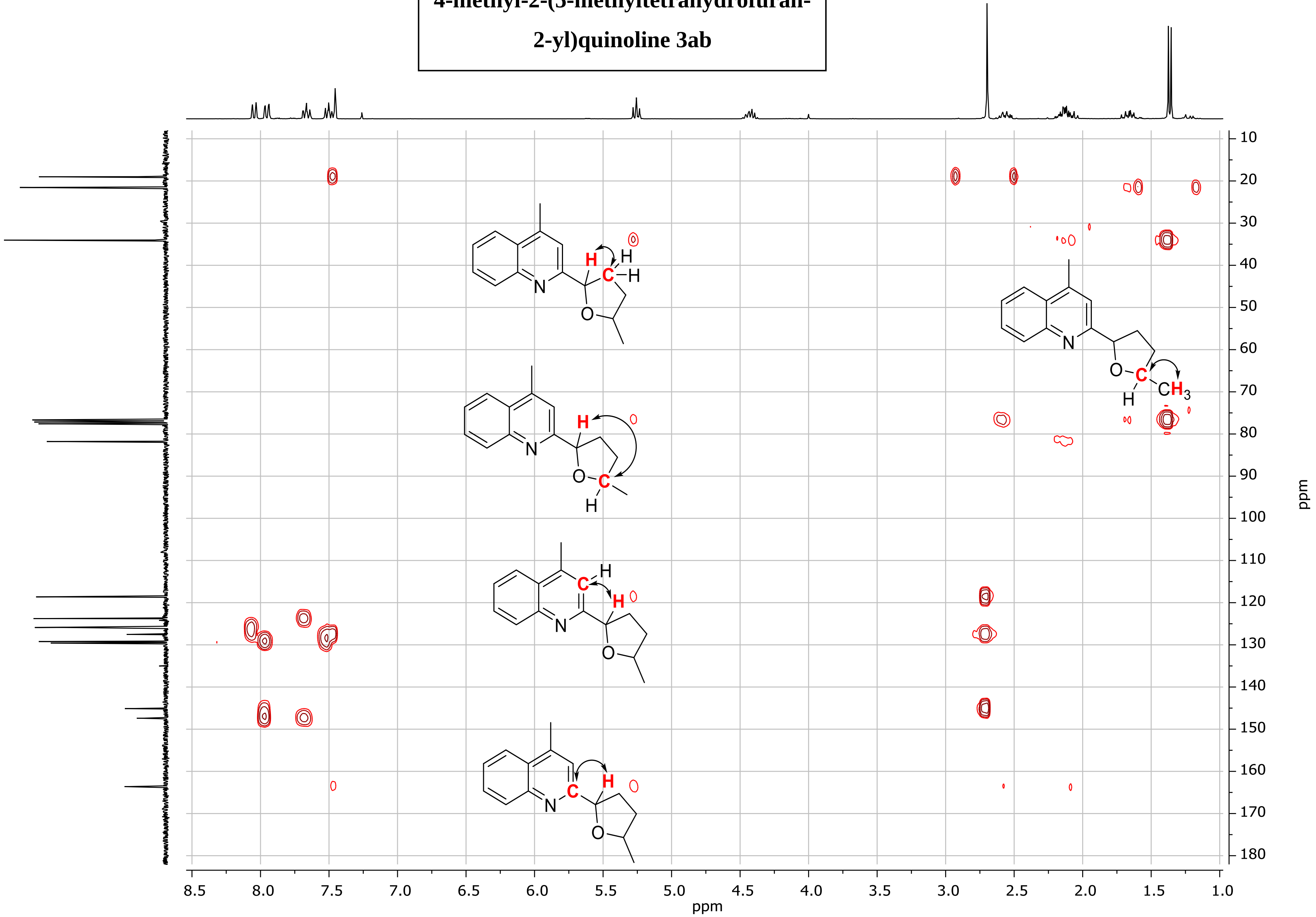

^{1}H-^{13}C HSQC

4-methyl-2-(5-methyltetrahydrofuran-2-yl)quinoline 3ab

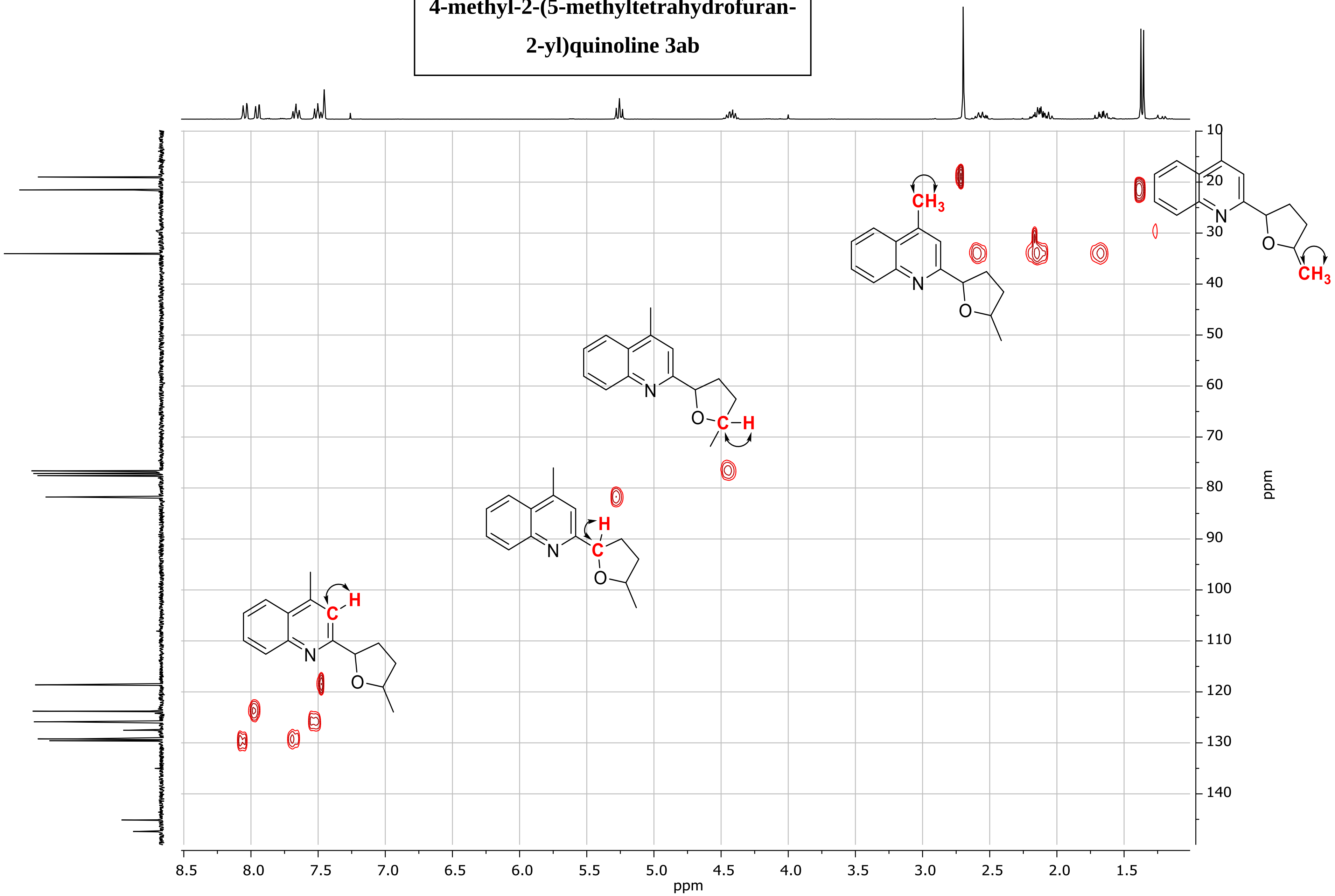

	Parameter	Value
1	Title	EL1001.{1H}.1.fid
2	Solvent	CDCl3
3	Temperature	298.0
4	Pulse Sequence	zg
5	Number of Scans	1
6	Receiver Gain	32.0
7	Relaxation Delay	0.1000
8	Pulse Width	14.7000
9	Acquisition Time	2.7724
10	Spectrometer Frequency	300.23
11	Spectral Width	5882.4
12	Lowest Frequency	-1094.5
13	Nucleus	1H
14	Acquired Size	16308
15	Spectral Size	65536

^{1}H NMR (300.13 MHz, $CDCl_3$)

4-methyl-2-(5-methyltetrahydrofuran-2-yl)quinoline 3ab

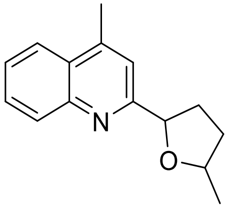

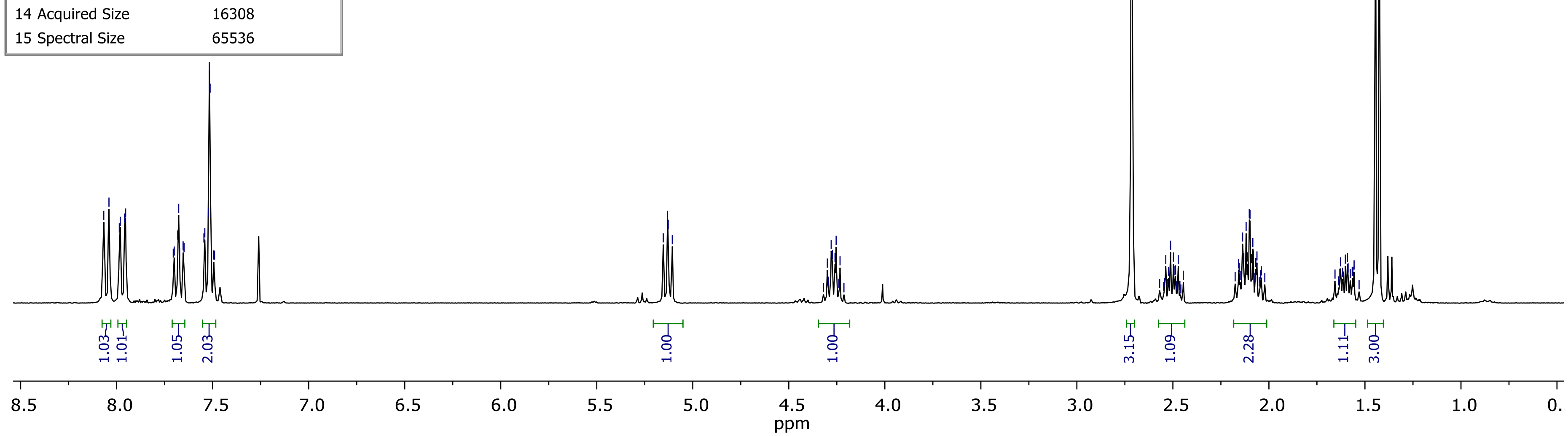

^{13}C NMR (75.48 MHz, $CDCl_3$)

4-methyl-2-(5-methyltetrahydrofuran-2-yl)quinoline 3ab

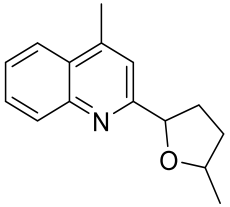

	Parameter	Value
1	Title	EL1001.{13C}.2.fid
2	Solvent	CDCl3
3	Temperature	298.0
4	Pulse Sequence	zgpg30
5	Number of Scans	300
6	Receiver Gain	101.0
7	Relaxation Delay	0.8000
8	Pulse Width	9.9000
9	Acquisition Time	1.1431
10	Spectrometer Frequency	75.50
11	Spectral Width	17857.1
12	Lowest Frequency	-1371.0
13	Nucleus	13C
14	Acquired Size	20412
15	Spectral Size	65536

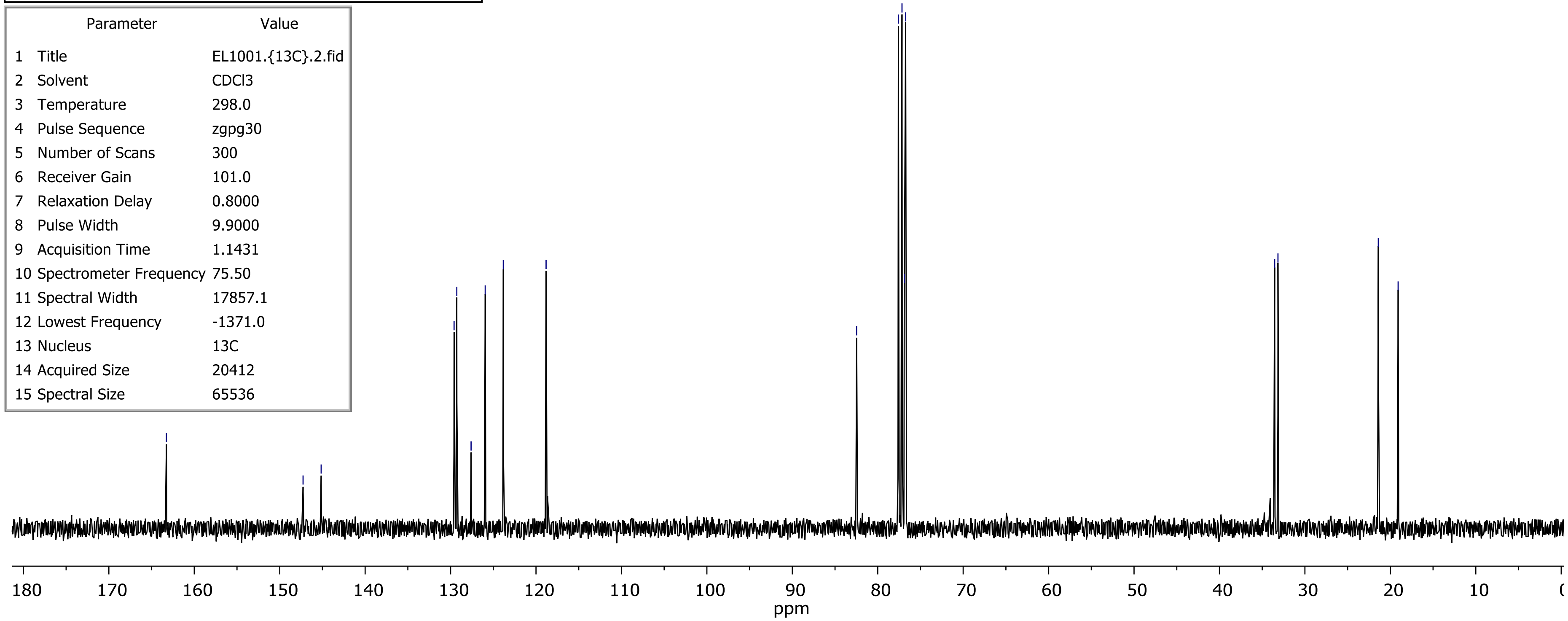

^{1}H-^{13}C HMBC

4-methyl-2-(5-methyltetrahydrofuran-2-yl)quinoline 3ab

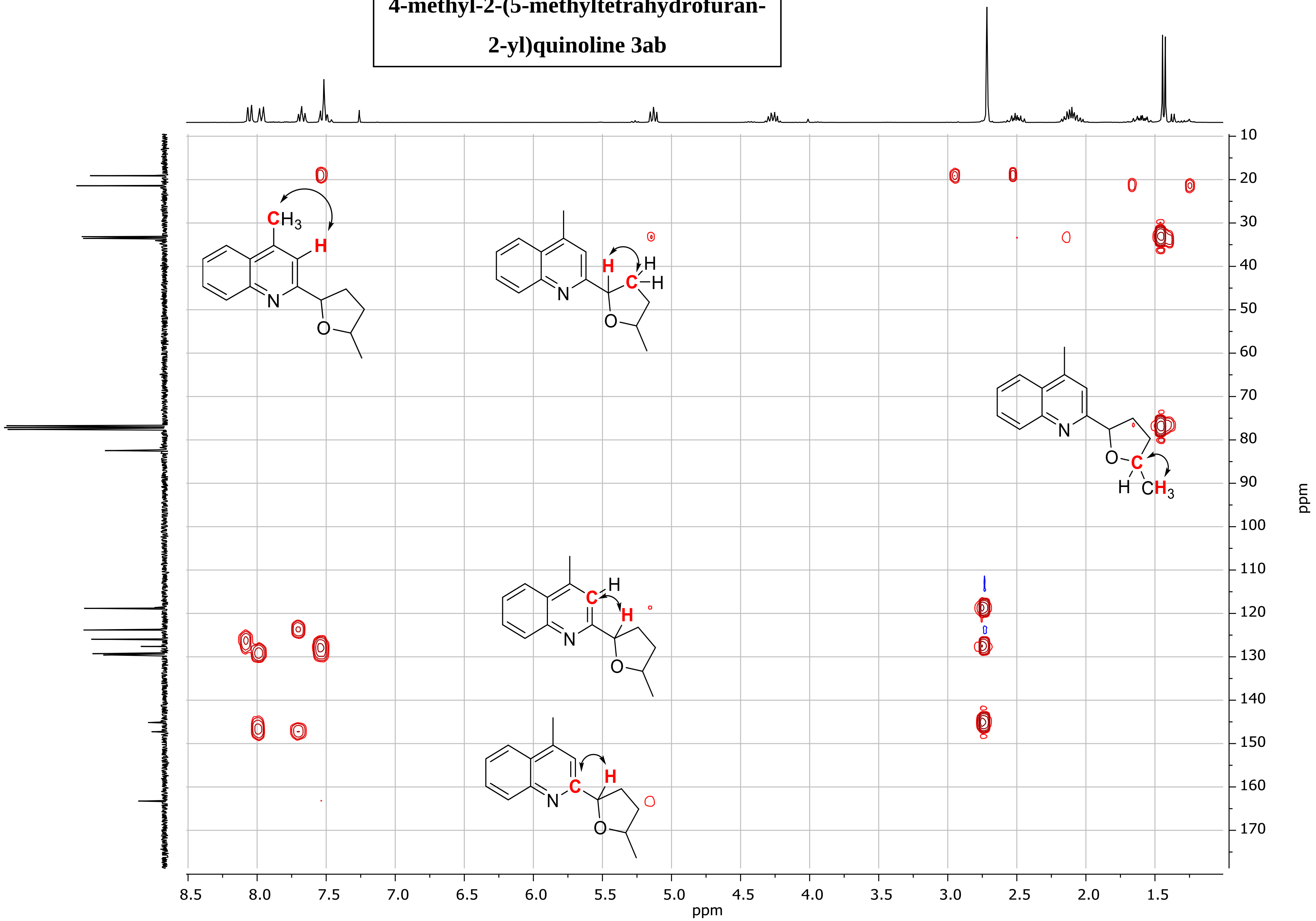

^1H-^{13}C HSQC

4-methyl-2-(5-methyltetrahydrofuran-2-yl)quinoline 3ab

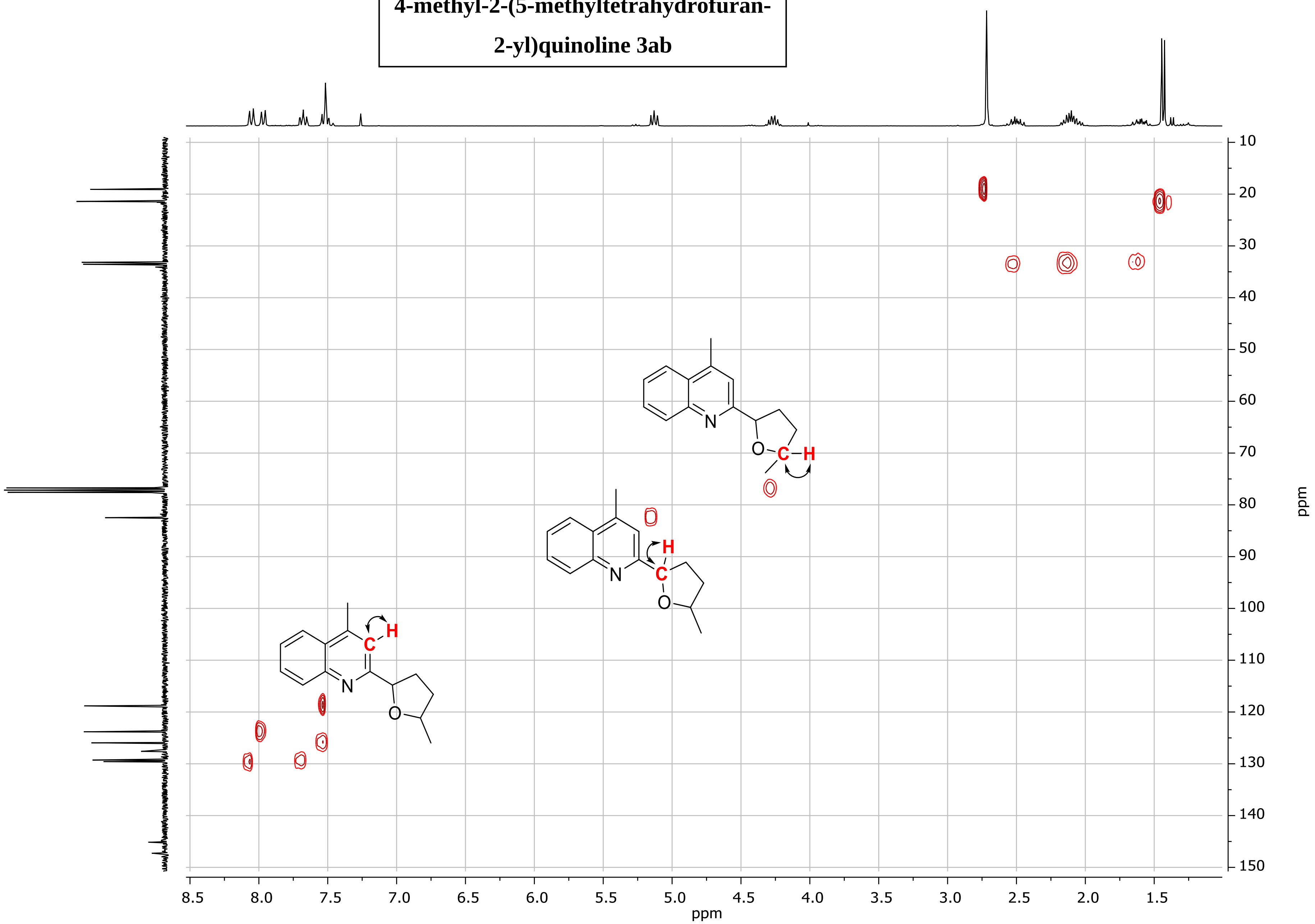

^{1}H NMR (300.13 MHz, $CDCl_3$)

4-methyl-2-(2-methyltetrahydrofuran-2-yl)quinoline 3ab’

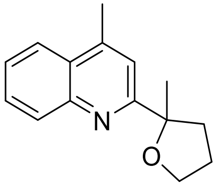

	Parameter	Value
1	Title	EL999.{1H}.1.fid
2	Solvent	CDCl3
3	Temperature	298.0
4	Pulse Sequence	zg
5	Number of Scans	1
6	Receiver Gain	32.0
7	Relaxation Delay	0.1000
8	Pulse Width	14.7000
9	Acquisition Time	2.7724
10	Spectrometer Frequency	300.23
11	Spectral Width	5882.4
12	Lowest Frequency	-1087.3
13	Nucleus	1H
14	Acquired Size	16308
15	Spectral Size	65536

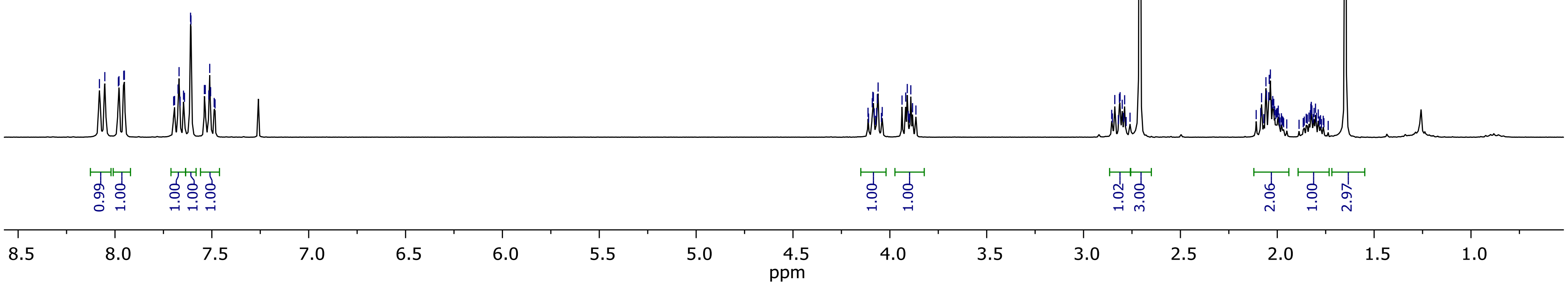

^{13}C NMR (75.48 MHz, $CDCl_3$)

4-methyl-2-(2-methyltetrahydrofuran-2-yl)quinoline 3ab'

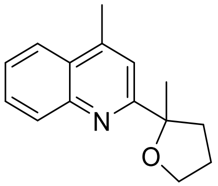

Parameter	Value
1 Title	EL999.{13C}.2.fid
2 Solvent	CDCl3
3 Temperature	298.0
4 Pulse Sequence	zgpg30
5 Number of Scans	300
6 Receiver Gain	101.0
7 Relaxation Delay	0.8000
8 Pulse Width	9.9000
9 Acquisition Time	1.1431
10 Spectrometer Frequency	75.50
11 Spectral Width	17857.1
12 Lowest Frequency	-1370.8
13 Nucleus	13C
14 Acquired Size	20412
15 Spectral Size	65536

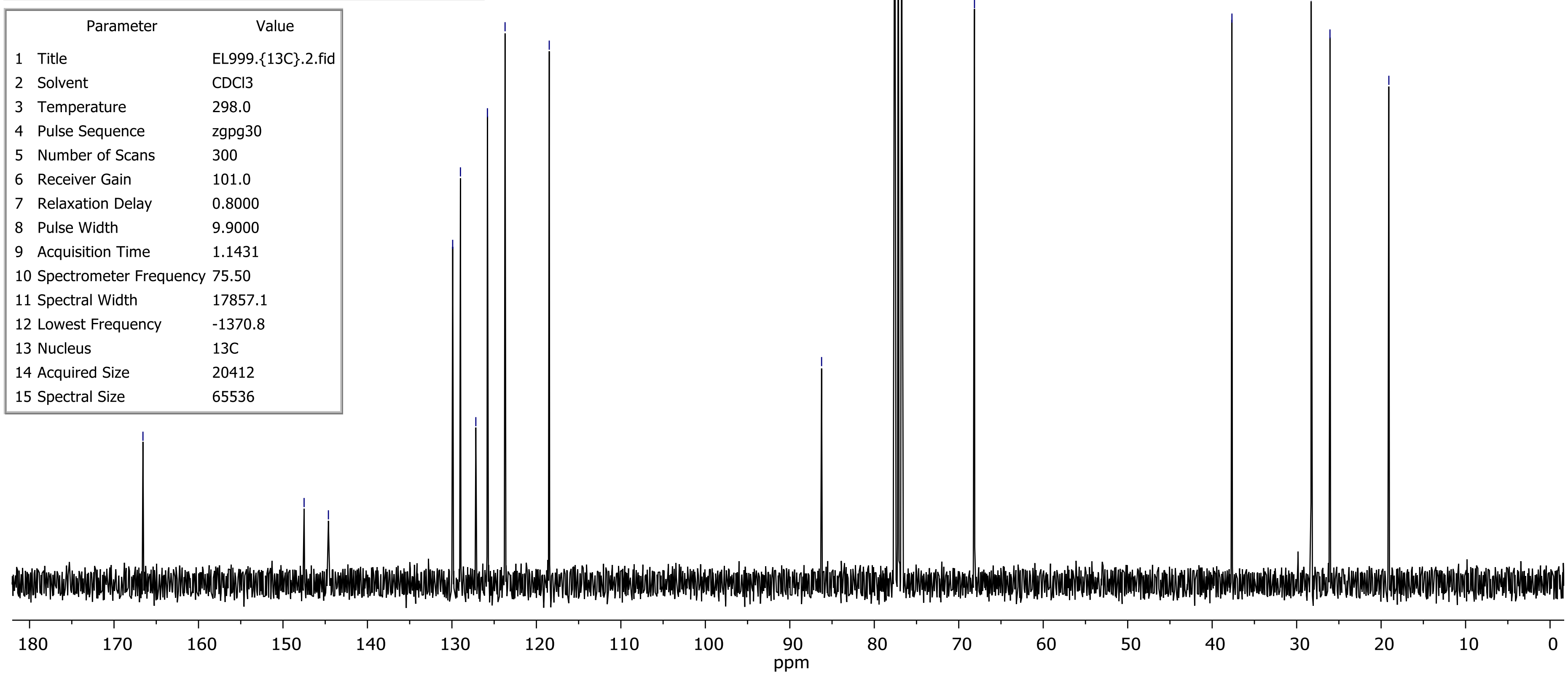

^{1}H-^{13}C HMBC

4-methyl-2-(2-methyltetrahydrofuran-2-yl)quinoline 3ab’

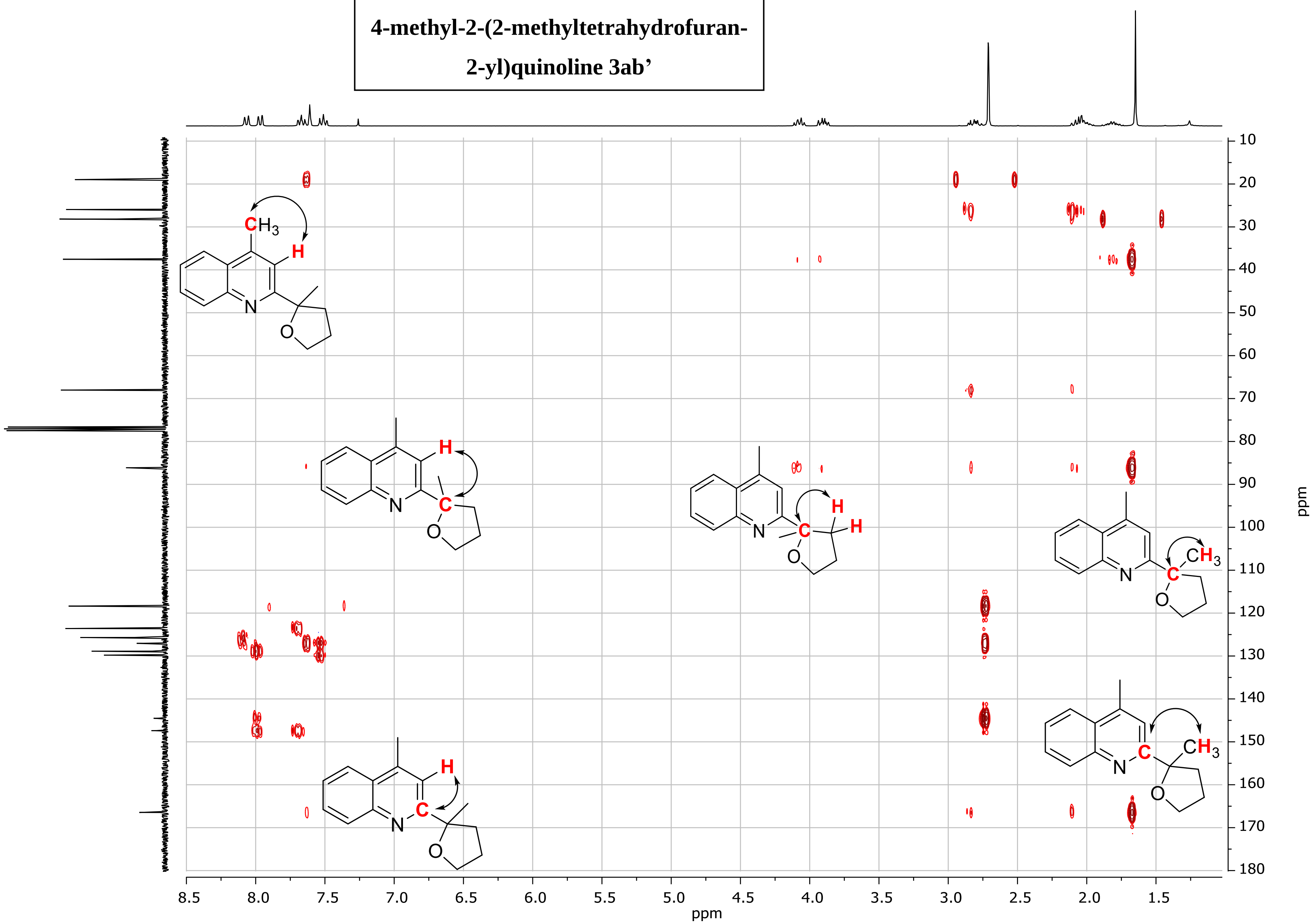

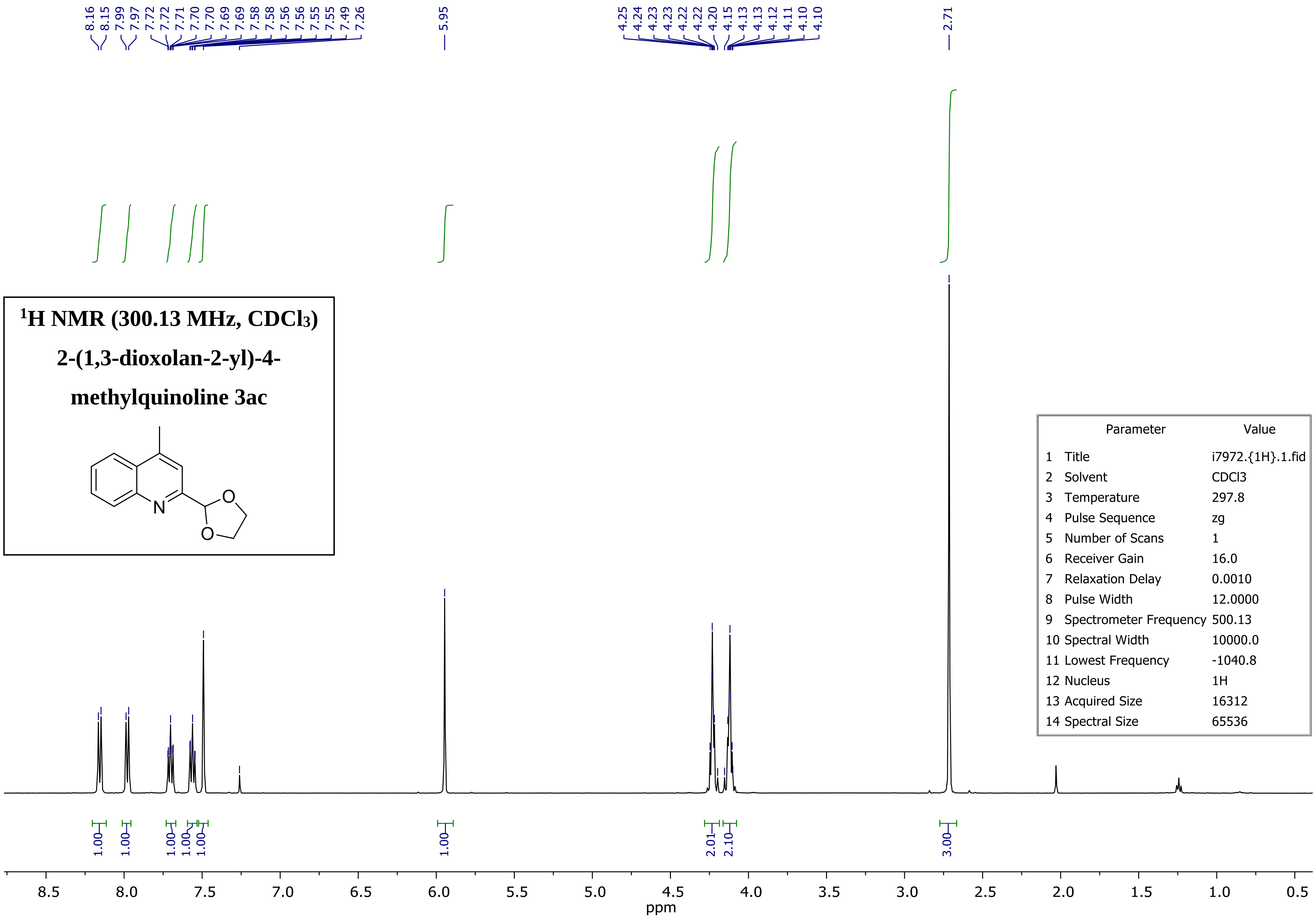

	Parameter	Value
1	Title	i7972.{1H}.1.fid
2	Solvent	CDCl3
3	Temperature	297.8
4	Pulse Sequence	zg
5	Number of Scans	1
6	Receiver Gain	16.0
7	Relaxation Delay	0.0010
8	Pulse Width	12.0000
9	Spectrometer Frequency	500.13
10	Spectral Width	10000.0
11	Lowest Frequency	-1040.8
12	Nucleus	1H
13	Acquired Size	16312
14	Spectral Size	65536

^{13}C NMR (75.48 MHz, $CDCl_3$)

2-(1,3-dioxolan-2-yl)-4-methylquinoline 3ac

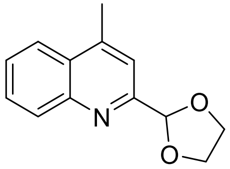

	Parameter	Value
1	Title	i7972.{13C}.1.fid
2	Solvent	CDCl3
3	Temperature	298.1
4	Pulse Sequence	zgdc
5	Number of Scans	33
6	Receiver Gain	5160.6
7	Relaxation Delay	2.0000
8	Pulse Width	6.3000
9	Spectrometer Frequency	125.77
10	Spectral Width	32679.7
11	Lowest Frequency	-2489.5
12	Nucleus	13C
13	Acquired Size	16325
14	Spectral Size	65536

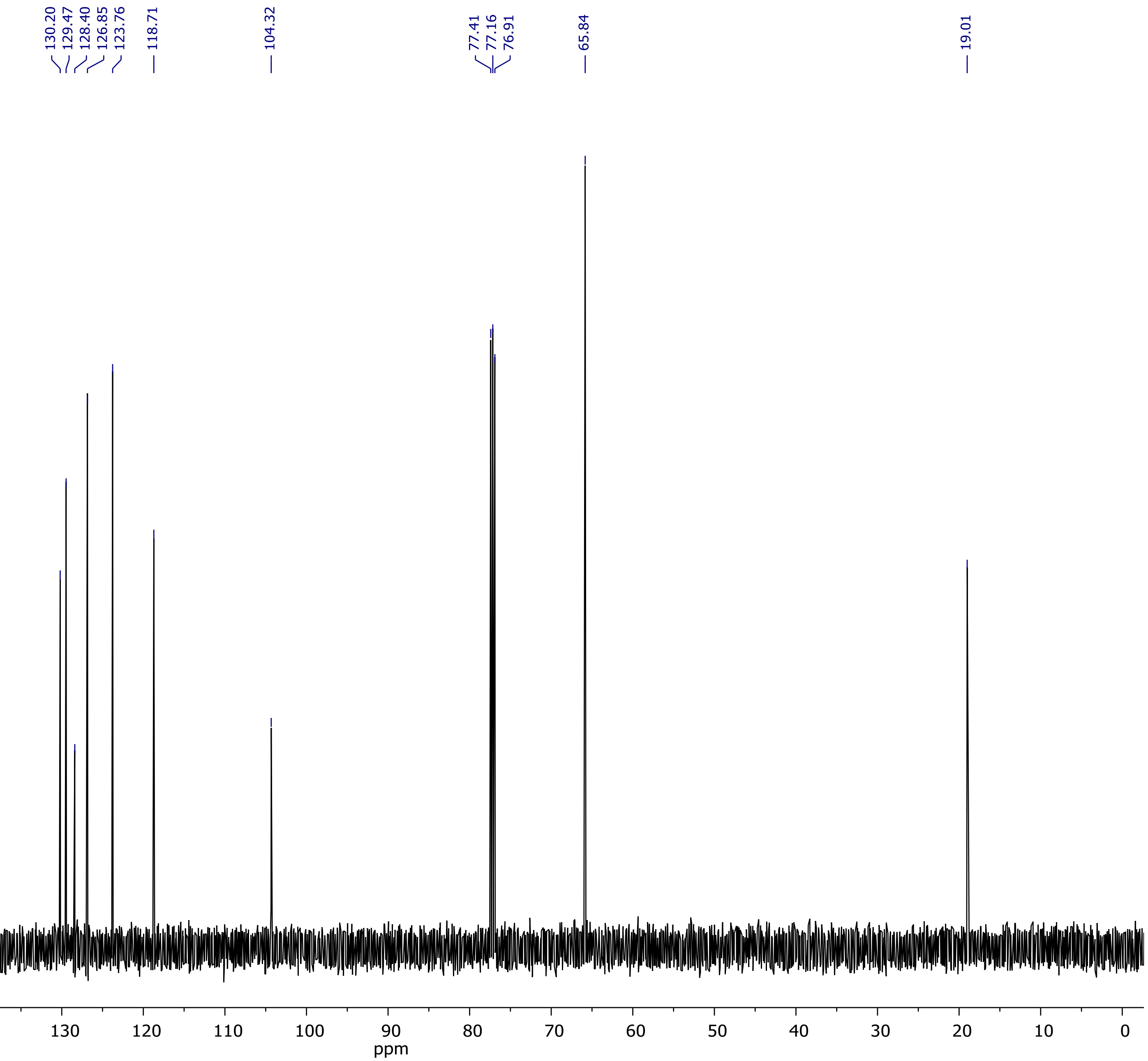

^{1}H NMR (300.13 MHz, $CDCl_3$)

2-(1,3-dioxolan-4-yl)-4-methylquinoline 3ac’

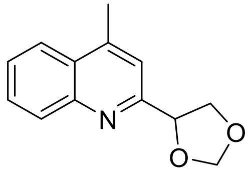

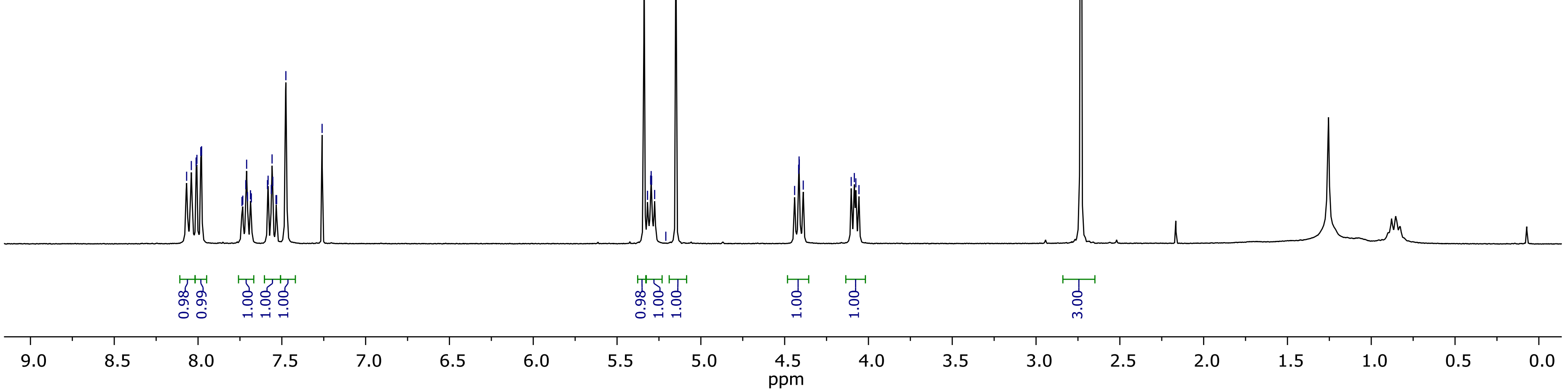

	Parameter	Value
1	Title	EL106.{1H}.1.fid
2	Solvent	CDCl3
3	Temperature	298.0
4	Pulse Sequence	zg
5	Number of Scans	1
6	Receiver Gain	32.0
7	Relaxation Delay	0.1000
8	Pulse Width	14.7000
9	Spectrometer Frequency	300.23
10	Spectral Width	5882.4
11	Lowest Frequency	-1094.5
12	Nucleus	1H
13	Acquired Size	16308
14	Spectral Size	65536

EL106-1.{13C}.1.fid
/TERN EL106-1

^{13}C NMR (75.48 MHz, $CDCl_3$)

2-(1,3-dioxolan-4-yl)-4-methylquinoline 3ac’

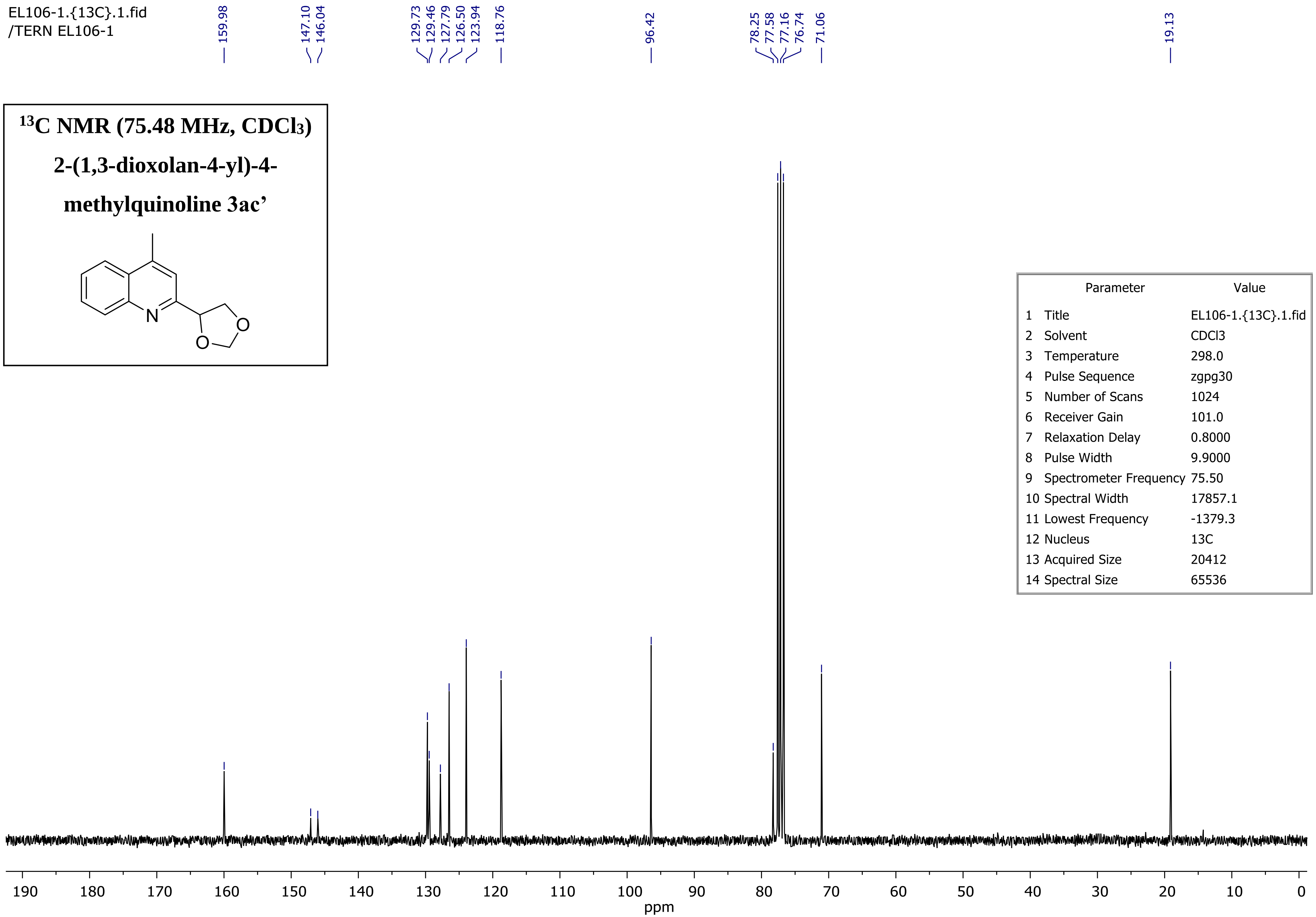

	Parameter	Value
1	Title	EL106-1.{13C}.1.fid
2	Solvent	CDCl3
3	Temperature	298.0
4	Pulse Sequence	zgpg30
5	Number of Scans	1024
6	Receiver Gain	101.0
7	Relaxation Delay	0.8000
8	Pulse Width	9.9000
9	Spectrometer Frequency	75.50
10	Spectral Width	17857.1
11	Lowest Frequency	-1379.3
12	Nucleus	13C
13	Acquired Size	20412
14	Spectral Size	65536

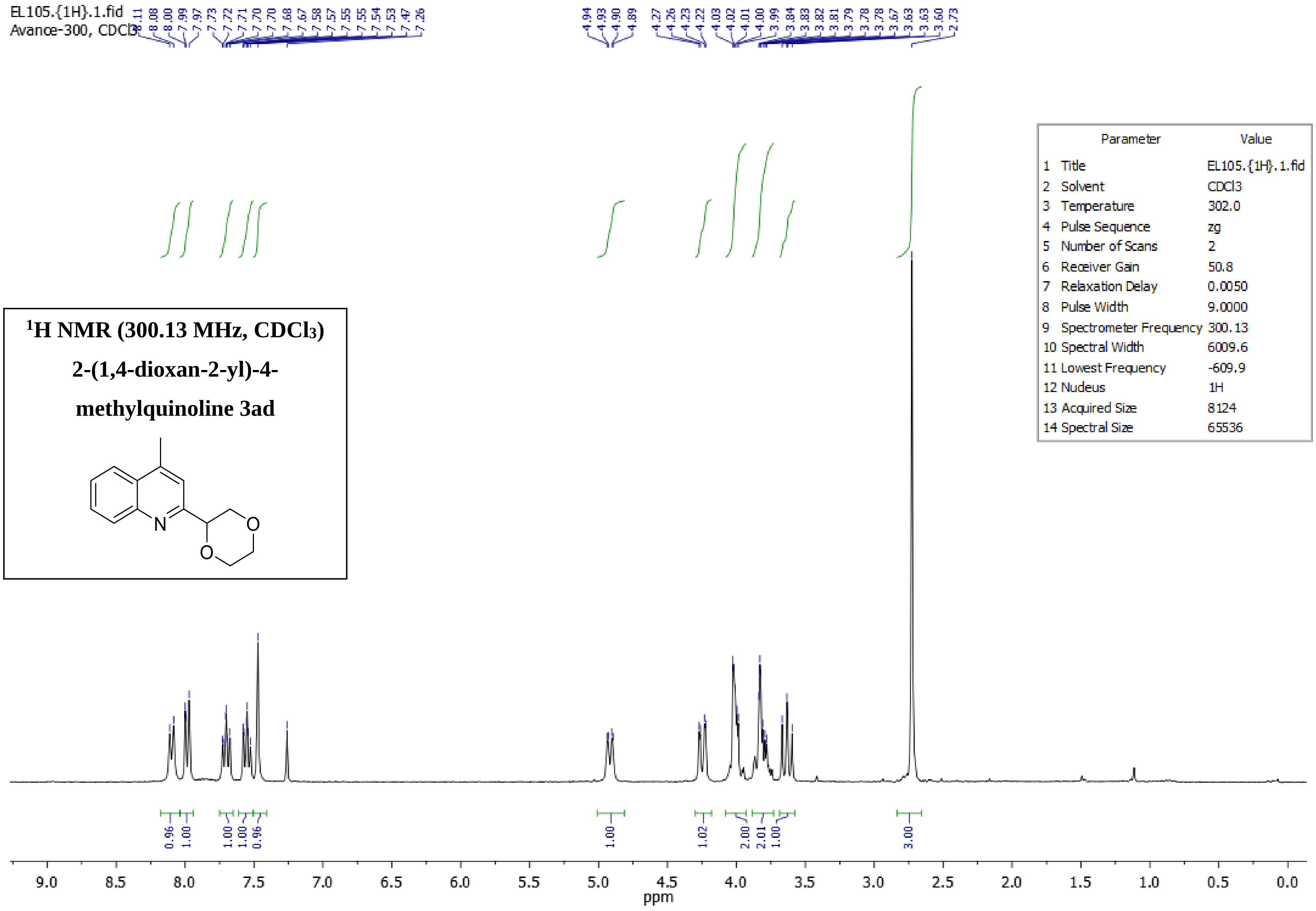
EL105.{1H}.1.fid
Avance-300, CDCl3
8.11 8.08 8.00 7.99 7.97 7.73 7.72 7.71 7.70 7.70 7.68 7.67 7.58 7.57 7.55 7.55 7.54 7.53 7.47 7.26
4.94 4.93 4.90 4.89 4.27 4.26 4.23 4.22 4.03 4.02 4.01 4.00 3.99 3.84 3.83 3.82 3.81 3.79 3.78 3.78 3.67 3.63 3.63 3.60 2.73
1H NMR (300.13 MHz, CDCl3)
2-(1,4-dioxan-2-yl)-4-methylquinoline 3ad
N
O
O
Parameter Value
1 Title EL105.{1H}.1.fid
2 Solvent CDCl3
3 Temperature 302.0
4 Pulse Sequence zg
5 Number of Scans 2
6 Receiver Gain 50.8
7 Relaxation Delay 0.0050
8 Pulse Width 9.0000
9 Spectrometer Frequency 300.13
10 Spectral Width 6009.6
11 Lowest Frequency -609.9
12 Nucleus 1H
13 Acquired Size 8124
14 Spectral Size 65536
0.96 1.00 1.00 1.00 0.96 1.00 1.02 2.00 2.01 1.00 3.00
9.0 8.5 8.0 7.5 7.0 6.5 6.0 5.5 5.0 4.5 4.0 3.5 3.0 2.5 2.0 1.5 1.0 0.5 0.0
ppm

^{13}C NMR (75.48 MHz, $CDCl_3$)

2-(1,4-dioxan-2-yl)-4-methylquinoline 3ad

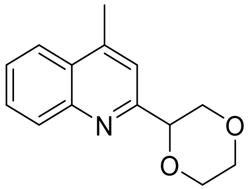

	Parameter	Value
1	Title	EL1006.{13C}.2.fid
2	Solvent	CDCl3
3	Temperature	298.0
4	Pulse Sequence	zgpg30
5	Number of Scans	350
6	Receiver Gain	101.0
7	Relaxation Delay	0.8000
8	Pulse Width	9.9000
9	Acquisition Time	1.1431
10	Spectrometer Frequency	75.50
11	Spectral Width	17857.1
12	Lowest Frequency	-1379.3
13	Nucleus	13C
14	Acquired Size	20412
15	Spectral Size	65536

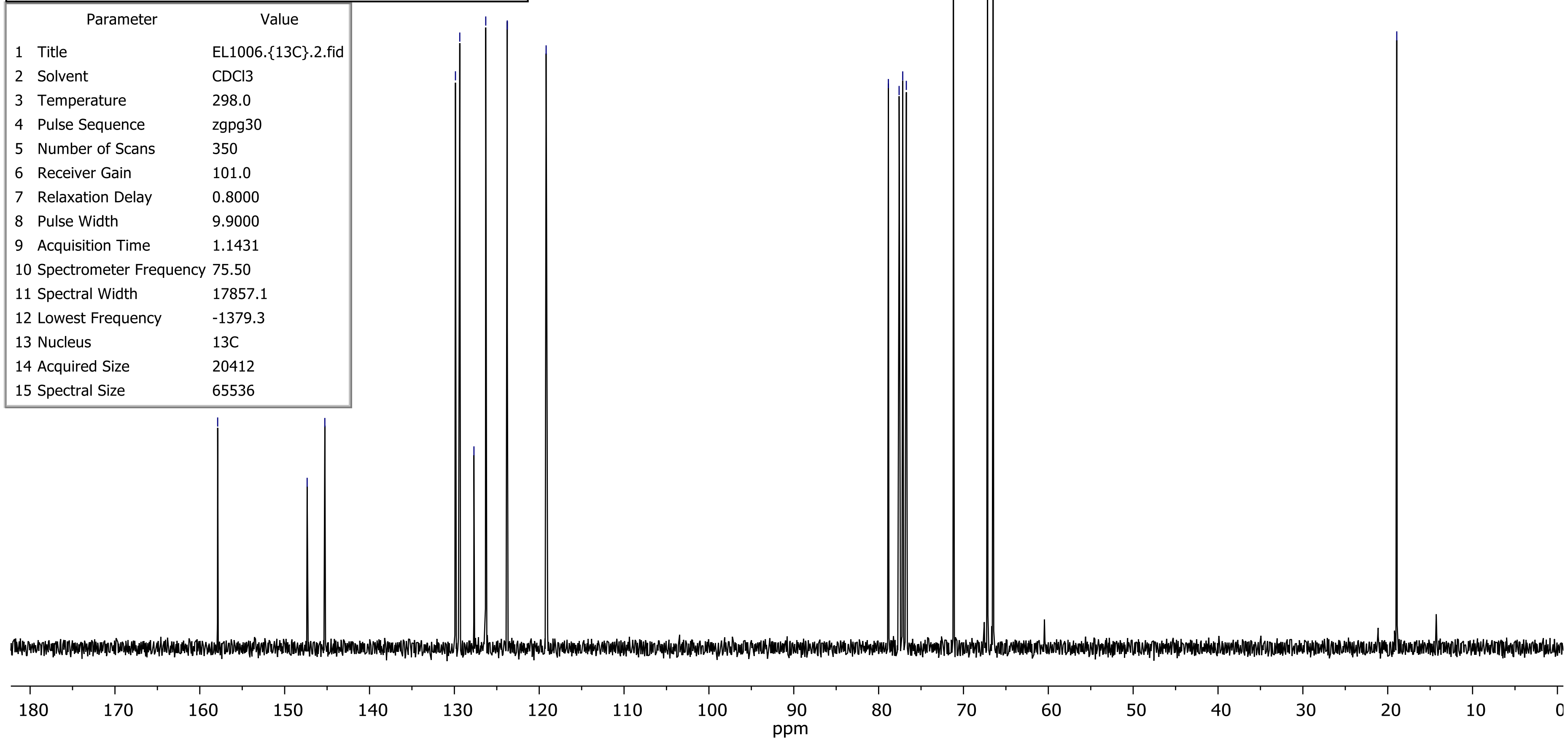

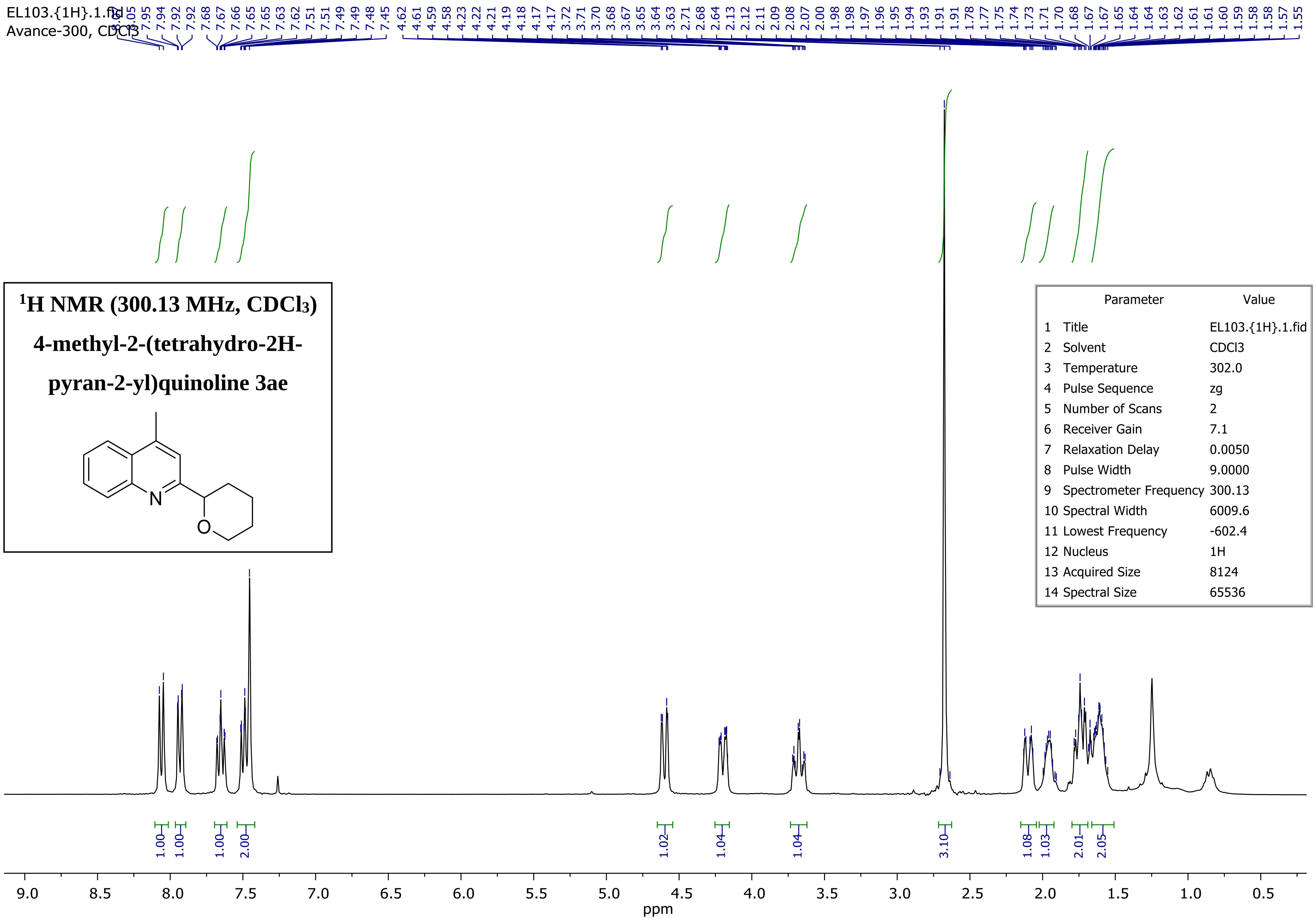

	Parameter	Value
1	Title	EL103.{1H}.1.fid
2	Solvent	CDCl3
3	Temperature	302.0
4	Pulse Sequence	zg
5	Number of Scans	2
6	Receiver Gain	7.1
7	Relaxation Delay	0.0050
8	Pulse Width	9.0000
9	Spectrometer Frequency	300.13
10	Spectral Width	6009.6
11	Lowest Frequency	-602.4
12	Nucleus	1H
13	Acquired Size	8124
14	Spectral Size	65536

EL103.{13C}.1.fid
EL103 Terentyev-04417

^{13}C NMR (75.48 MHz, $CDCl_3$)

4-methyl-2-(tetrahydro-2H-pyran-2-yl)quinoline 3ae

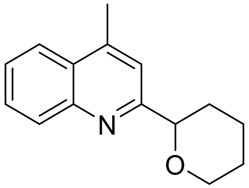

Parameter		Value
1	Title	EL103.{13C}.1.fid
2	Solvent	CDCl3
3	Temperature	300.0
4	Pulse Sequence	zgpg30
5	Number of Scans	100
6	Receiver Gain	214.2
7	Relaxation Delay	0.8000
8	Pulse Width	9.3000
9	Spectrometer Frequency	75.48
10	Spectral Width	18028.8
11	Lowest Frequency	-1464.8
12	Nucleus	13C
13	Acquired Size	16316
14	Spectral Size	65536

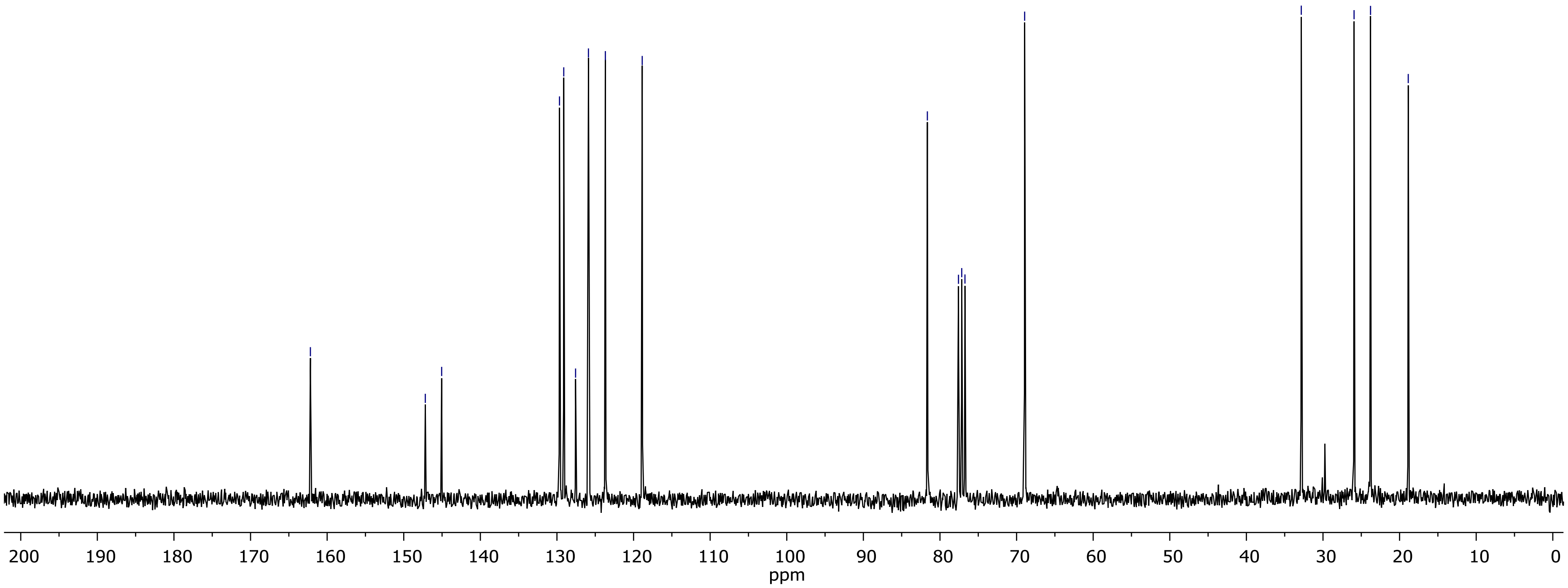

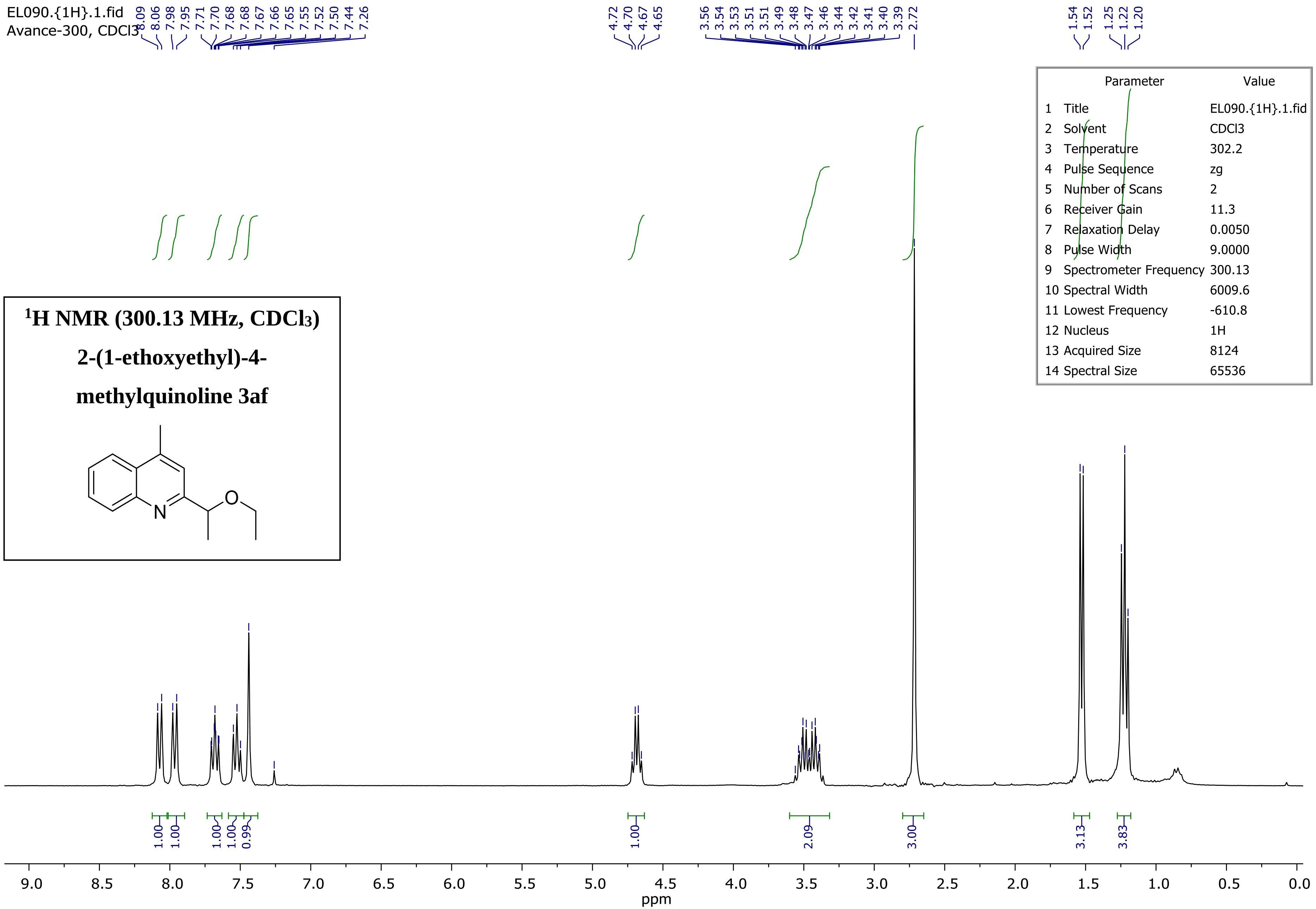

	Parameter	Value
1	Title	EL090.{1H}.1.fid
2	Solvent	CDCl3
3	Temperature	302.2
4	Pulse Sequence	zg
5	Number of Scans	2
6	Receiver Gain	11.3
7	Relaxation Delay	0.0050
8	Pulse Width	9.0000
9	Spectrometer Frequency	300.13
10	Spectral Width	6009.6
11	Lowest Frequency	-610.8
12	Nucleus	1H
13	Acquired Size	8124
14	Spectral Size	65536

EL090.{13C}.13.fid
/LB58 IYT1588

^{13}C NMR (75.48 MHz, $CDCl_3$)

2-(1-ethoxyethyl)-4-methylquinoline 3af

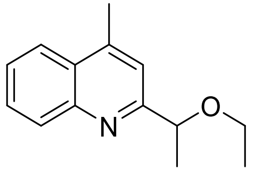

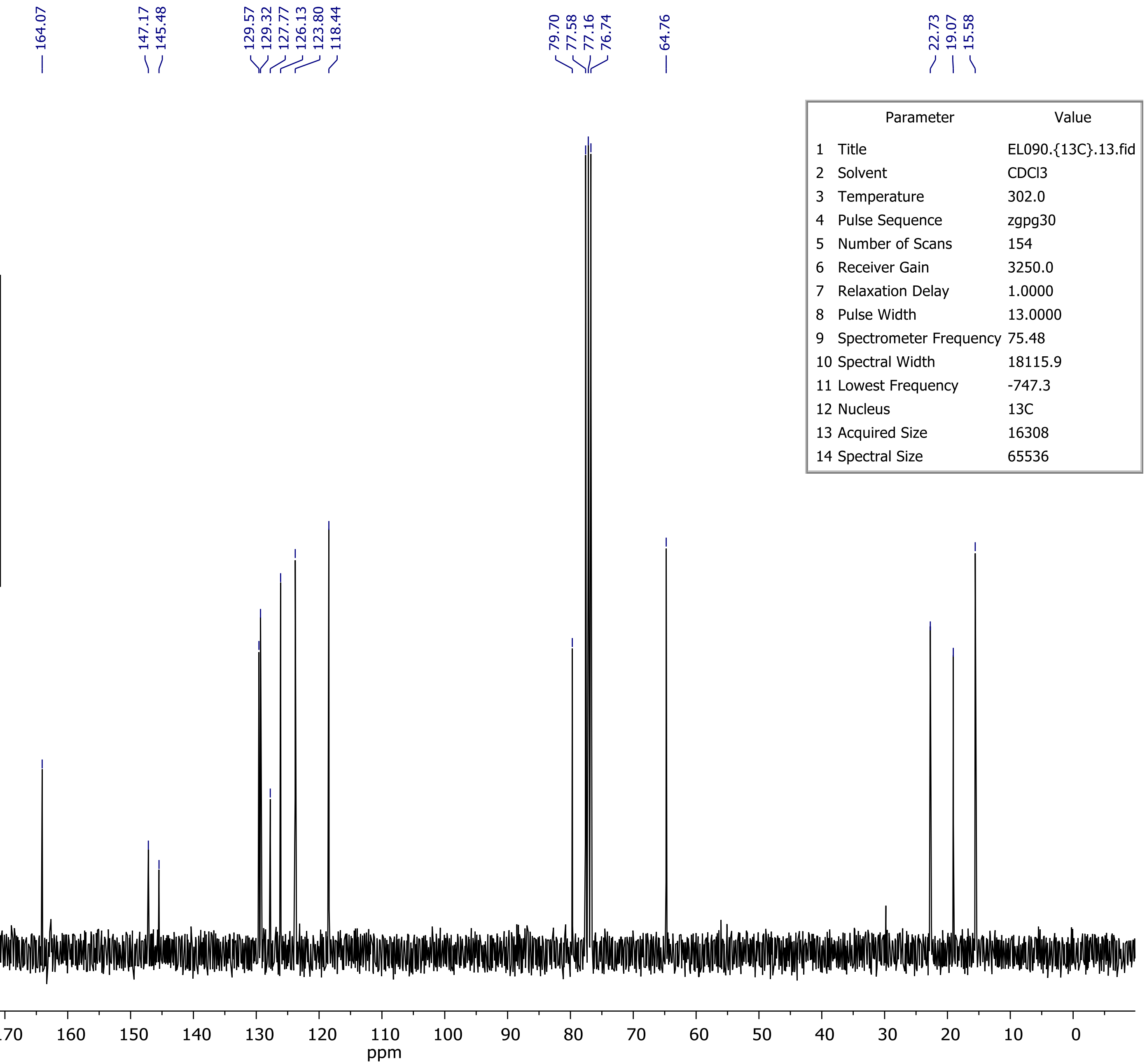

	Parameter	Value
1	Title	EL090.{13C}.13.fid
2	Solvent	CDCl3
3	Temperature	302.0
4	Pulse Sequence	zgpg30
5	Number of Scans	154
6	Receiver Gain	3250.0
7	Relaxation Delay	1.0000
8	Pulse Width	13.0000
9	Spectrometer Frequency	75.48
10	Spectral Width	18115.9
11	Lowest Frequency	-747.3
12	Nucleus	13C
13	Acquired Size	16308
14	Spectral Size	65536

^{1}H NMR (300.13 MHz, $CDCl_3$)

2-methoxy-4-(tetrahydrofuran-2-yl)quinoline 3ba

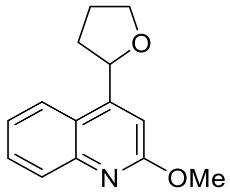

	Parameter	Value
1	Title	i8820.{1H}.1.fid
2	Solvent	CDCl3
3	Temperature	301.8
4	Pulse Sequence	zg
5	Number of Scans	4
6	Receiver Gain	5.0
7	Relaxation Delay	1.0000
8	Pulse Width	9.0000
9	Spectrometer Frequency	300.13
10	Spectral Width	6009.6
11	Lowest Frequency	-609.9
12	Nucleus	1H
13	Acquired Size	8124
14	Spectral Size	65536

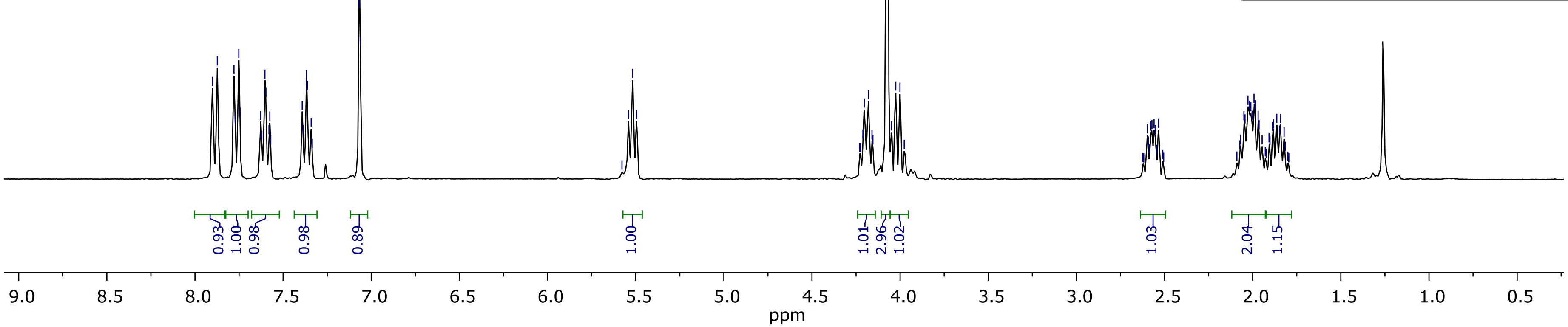

i8820.{13C}.13.fid
/LB58 IYT1588

^{13}C NMR (75.48 MHz, $CDCl_3$)

2-methoxy-4-(tetrahydrofuran-2-yl)quinoline 3ba

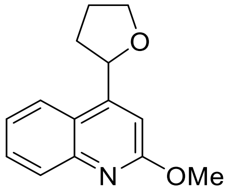

Parameter		Value
1	Title	i8820.{13C}.13.fid
2	Solvent	CDCl3
3	Temperature	302.0
4	Pulse Sequence	zgpg30
5	Number of Scans	230
6	Receiver Gain	3250.0
7	Relaxation Delay	1.0000
8	Pulse Width	13.0000
9	Spectrometer Frequency	75.48
10	Spectral Width	18115.9
11	Lowest Frequency	-747.7
12	Nucleus	13C
13	Acquired Size	16308
14	Spectral Size	65536

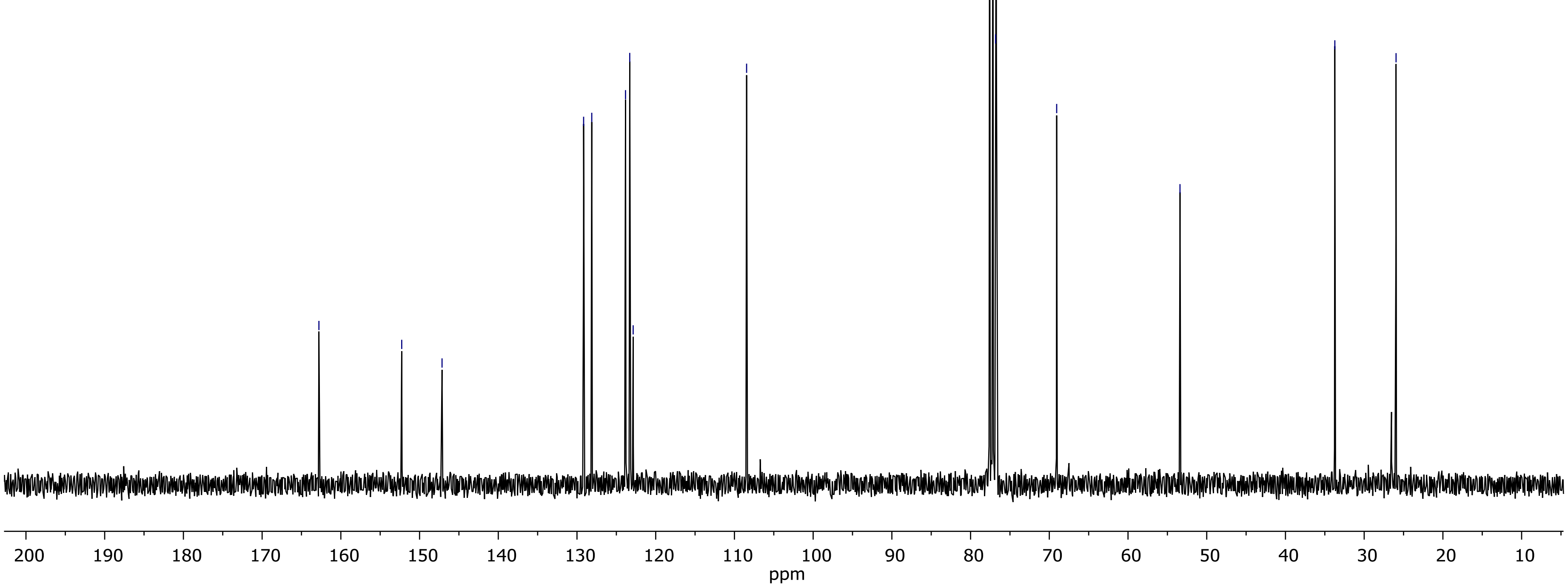

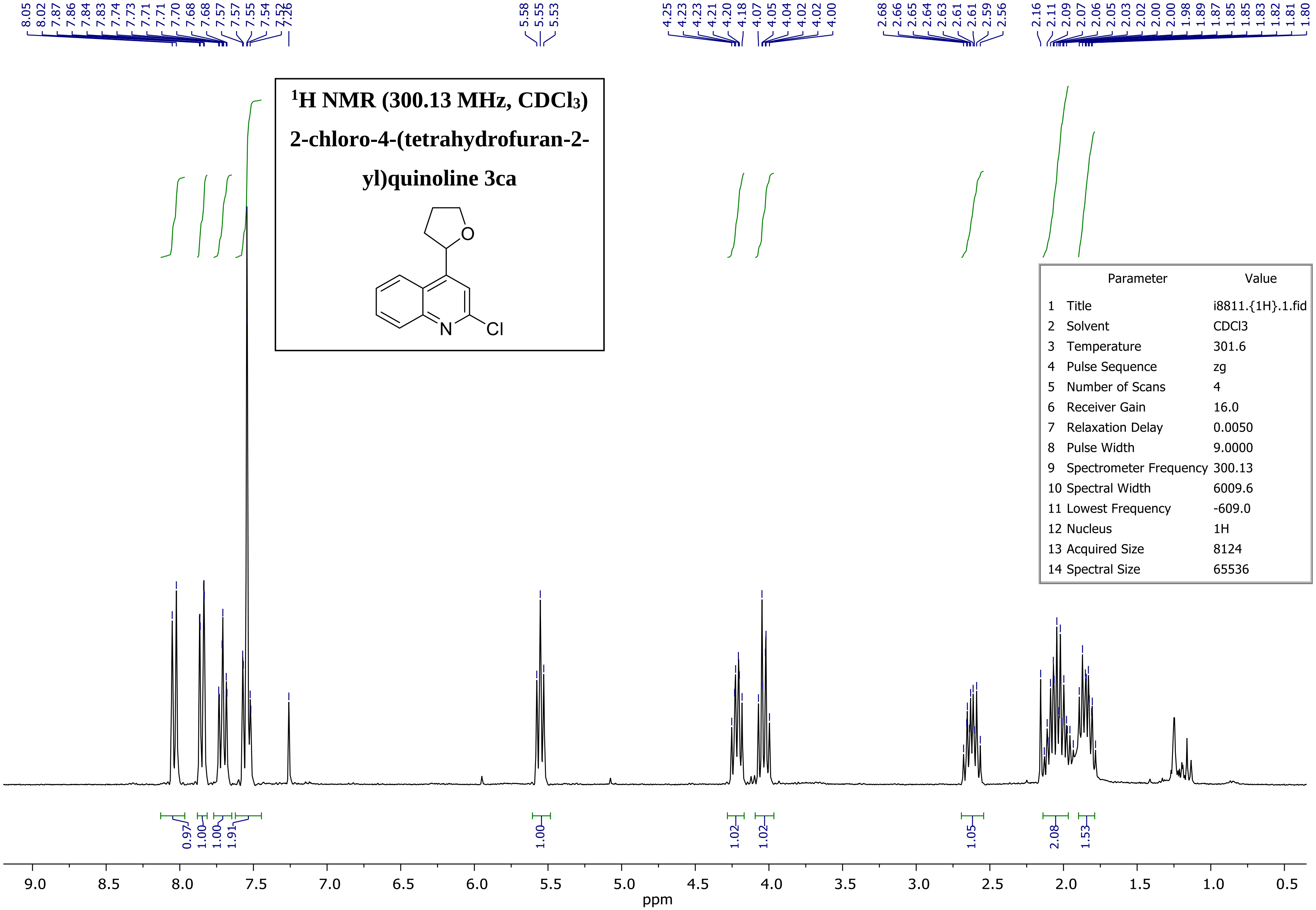

^{1}H NMR (300.13 MHz, $CDCl_3$)

2-chloro-4-(tetrahydrofuran-2-yl)quinoline 3ca

	Parameter	Value
1	Title	i8811.{1H}.1.fid
2	Solvent	CDCl3
3	Temperature	301.6
4	Pulse Sequence	zg
5	Number of Scans	4
6	Receiver Gain	16.0
7	Relaxation Delay	0.0050
8	Pulse Width	9.0000
9	Spectrometer Frequency	300.13
10	Spectral Width	6009.6
11	Lowest Frequency	-609.0
12	Nucleus	1H
13	Acquired Size	8124
14	Spectral Size	65536

i8811.{13C}.13.fid
/LB58 IYT1588

^{13}C NMR (75.48 MHz, $CDCl_3$)

2-chloro-4-(tetrahydrofuran-2-yl)quinoline 3ca

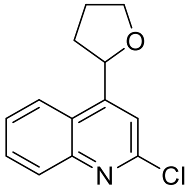

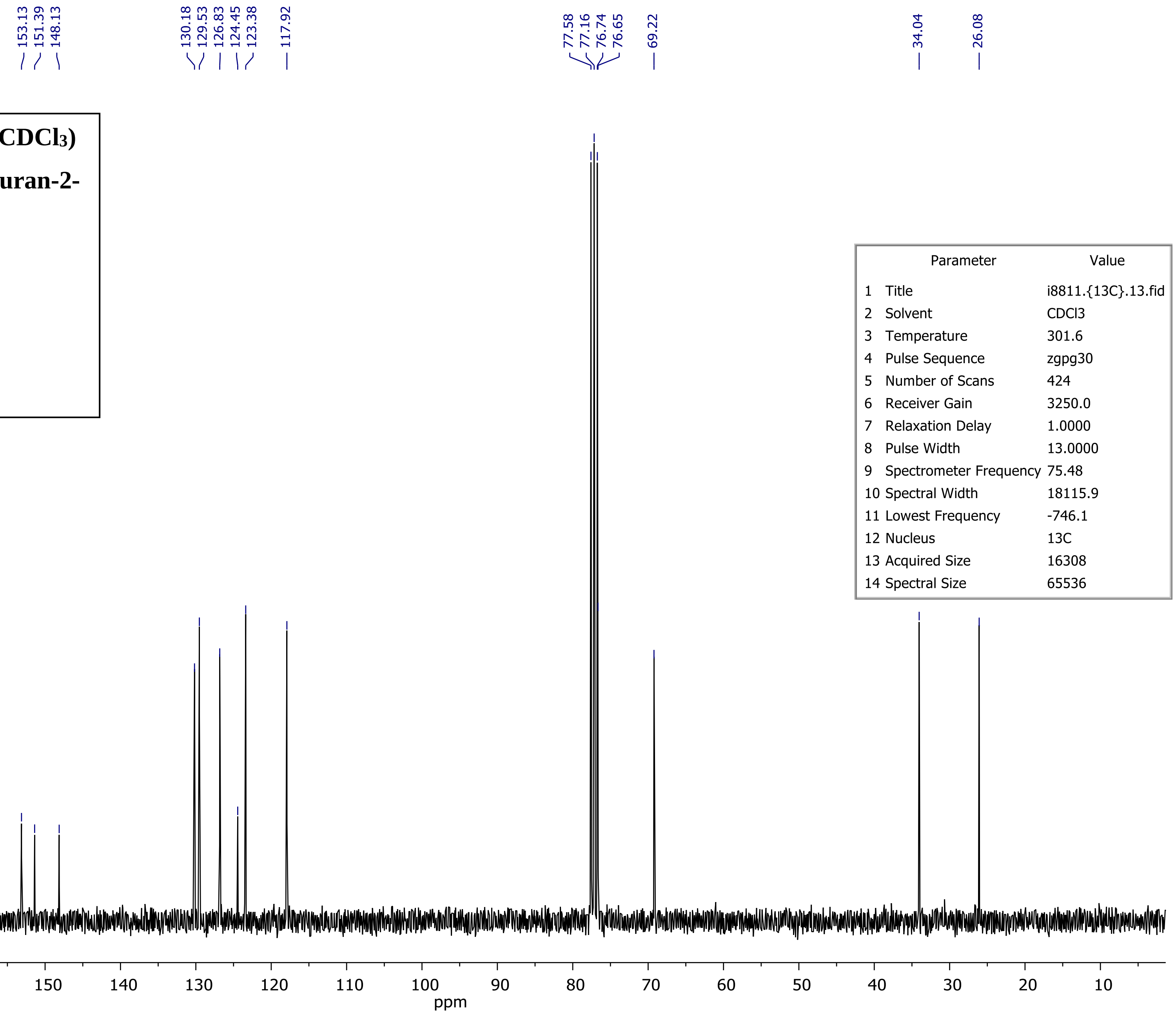

	Parameter	Value
1	Title	i8811.{13C}.13.fid
2	Solvent	CDCl3
3	Temperature	301.6
4	Pulse Sequence	zgpg30
5	Number of Scans	424
6	Receiver Gain	3250.0
7	Relaxation Delay	1.0000
8	Pulse Width	13.0000
9	Spectrometer Frequency	75.48
10	Spectral Width	18115.9
11	Lowest Frequency	-746.1
12	Nucleus	13C
13	Acquired Size	16308
14	Spectral Size	65536

Parameter		Value
1	Title	EL583.{1H}.1.fid
2	Solvent	CDCl3
3	Temperature	298.0
4	Pulse Sequence	zg
5	Number of Scans	1
6	Receiver Gain	32.0
7	Relaxation Delay	0.1000
8	Pulse Width	14.7000
9	Spectrometer Frequency	300.23
10	Spectral Width	5882.4
11	Lowest Frequency	-1094.5
12	Nucleus	1H
13	Acquired Size	16308
14	Spectral Size	65536

^{1}H NMR (300.13 MHz, $CDCl_3$)

2-methyl-4-(tetrahydrofuran-2-yl)quinoline 3ea

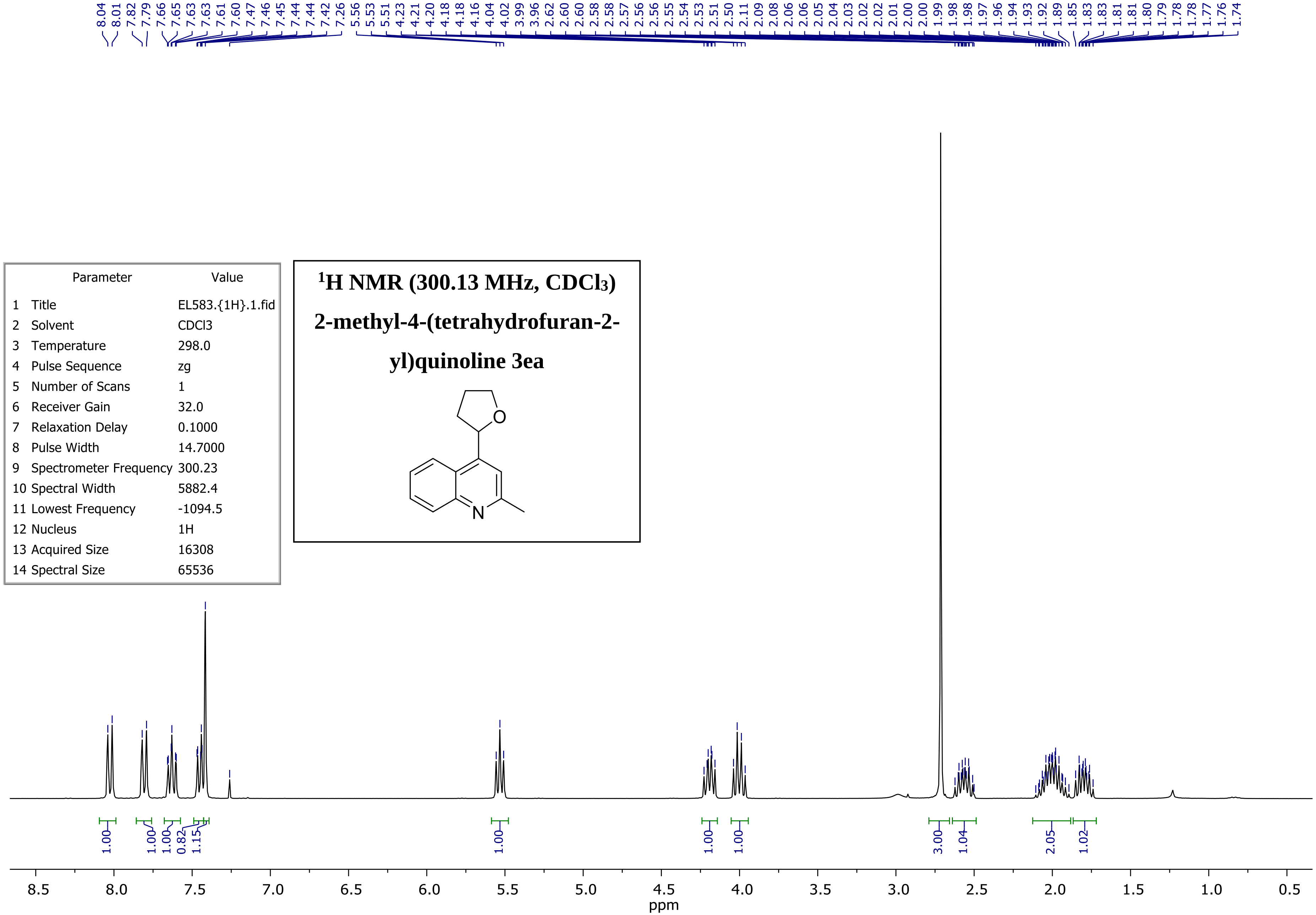

^{13}C NMR (75.48 MHz, CDCl$_3$)

2-methyl-4-(tetrahydrofuran-2-yl)quinoline 3ea

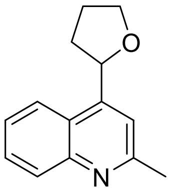

Parameter		Value
1	Title	EL583.{13C}.2.fid
2	Solvent	CDCl3
3	Temperature	298.0
4	Pulse Sequence	zgpg30
5	Number of Scans	300
6	Receiver Gain	101.0
7	Relaxation Delay	0.8000
8	Pulse Width	9.9000
9	Spectrometer Frequency	75.50
10	Spectral Width	17857.1
11	Lowest Frequency	-1376.1
12	Nucleus	13C
13	Acquired Size	20412
14	Spectral Size	65536

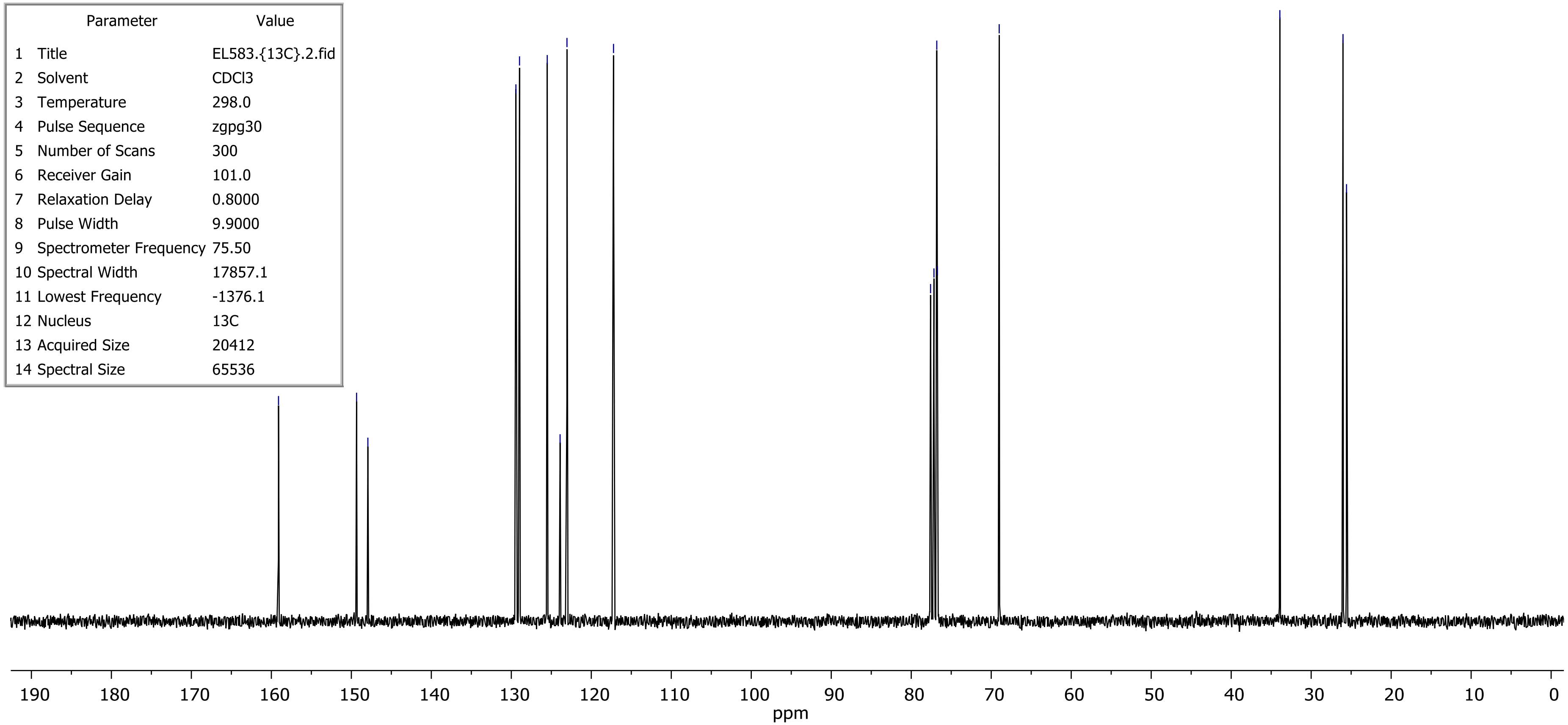

^{1}H NMR (300.13 MHz, $CDCl_3$)

2-(tetrahydrofuran-2-yl)quinoxaline 3fa

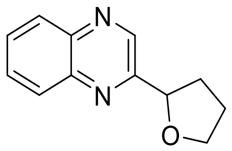

	Parameter	Value
1	Title	EL096.{1H}.1.fid
2	Solvent	CDCl3
3	Temperature	297.4
4	Pulse Sequence	zg
5	Number of Scans	1
6	Receiver Gain	16.0
7	Relaxation Delay	0.0010
8	Pulse Width	12.0000
9	Spectrometer Frequency	500.13
10	Spectral Width	10000.0
11	Lowest Frequency	-1004.3
12	Nucleus	1H
13	Acquired Size	16312
14	Spectral Size	65536

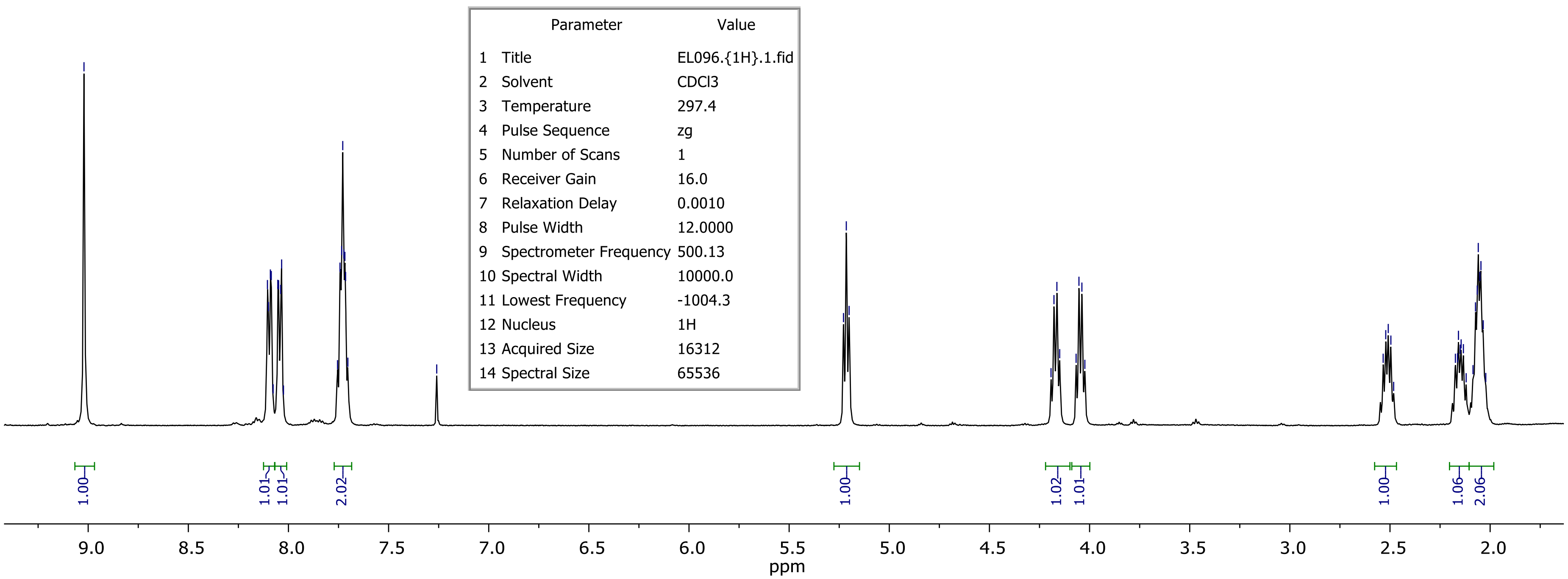

EL096.{13C}.1.fid
/TERN EL096

^{13}C NMR (75.48 MHz, $CDCl_3$)

2-(tetrahydrofuran-2-yl)quinoxaline 3fa

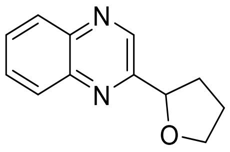

	Parameter	Value
1	Title	EL096.{13C}.1.fid
2	Solvent	CDCl3
3	Temperature	297.3
4	Pulse Sequence	zgdc
5	Number of Scans	407
6	Receiver Gain	5160.6
7	Relaxation Delay	2.0000
8	Pulse Width	6.3000
9	Spectrometer Frequency	125.77
10	Spectral Width	32679.7
11	Lowest Frequency	-2488.0
12	Nucleus	13C
13	Acquired Size	16325
14	Spectral Size	65536

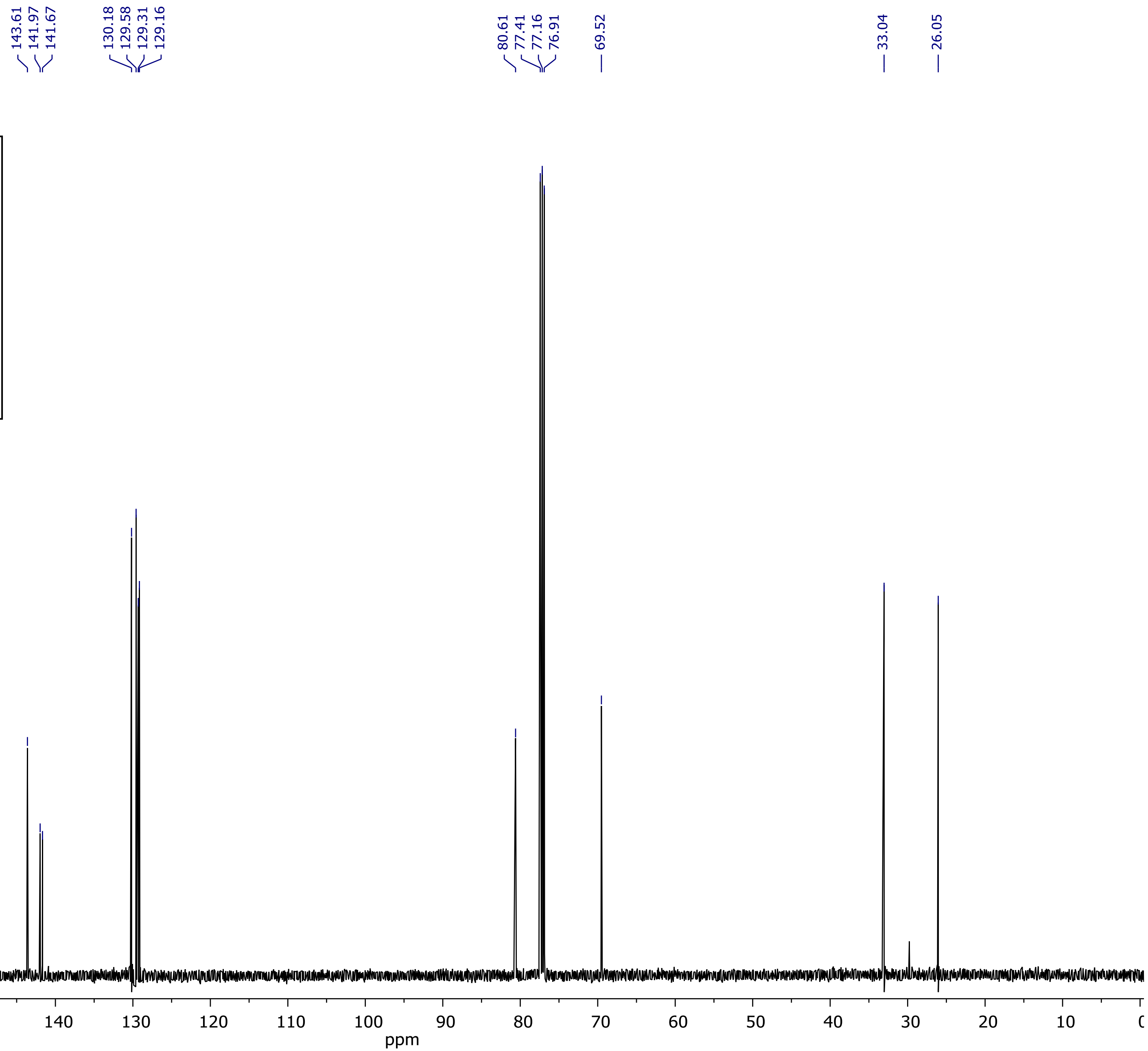

^{1}H NMR (300.13 MHz, $CDCl_3$)

2-(tetrahydrofuran-2-yl)pyrazine 3ga

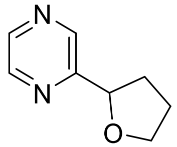

	Parameter	Value
1	Title	EL1351-1.{1H}.1.fid
2	Solvent	CDCl3
3	Temperature	298.0
4	Pulse Sequence	zg
5	Number of Scans	1
6	Receiver Gain	32.0
7	Relaxation Delay	2.0000
8	Pulse Width	14.7000
9	Acquisition Time	2.7724
10	Spectrometer Frequency	300.23
11	Spectral Width	5882.4
12	Lowest Frequency	-1094.5
13	Nucleus	1H
14	Acquired Size	16308
15	Spectral Size	65536

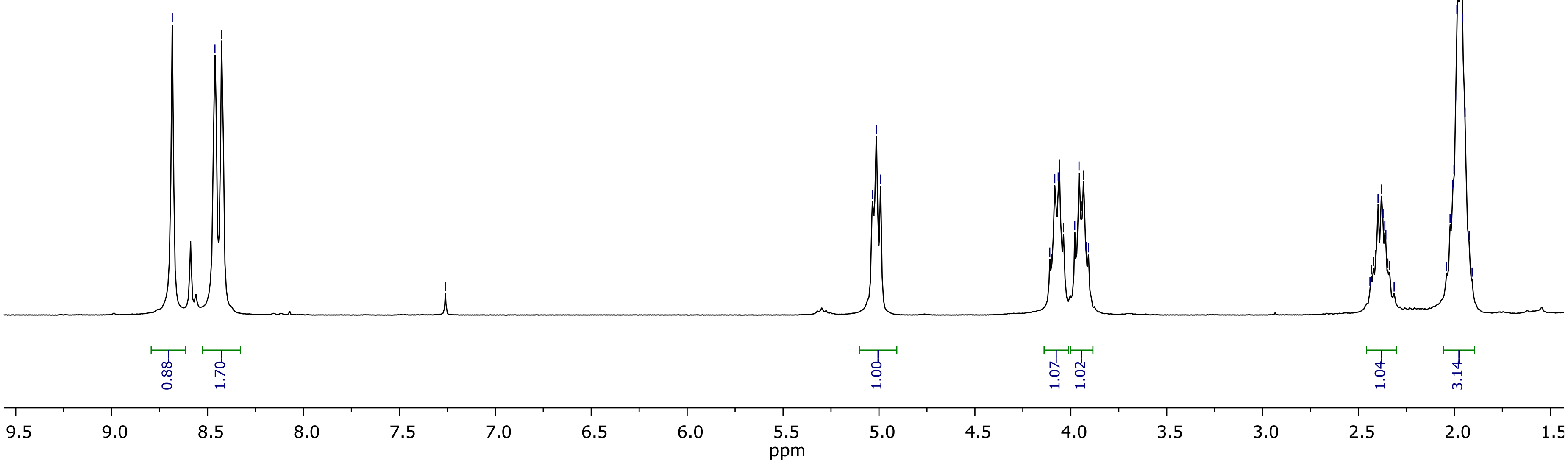

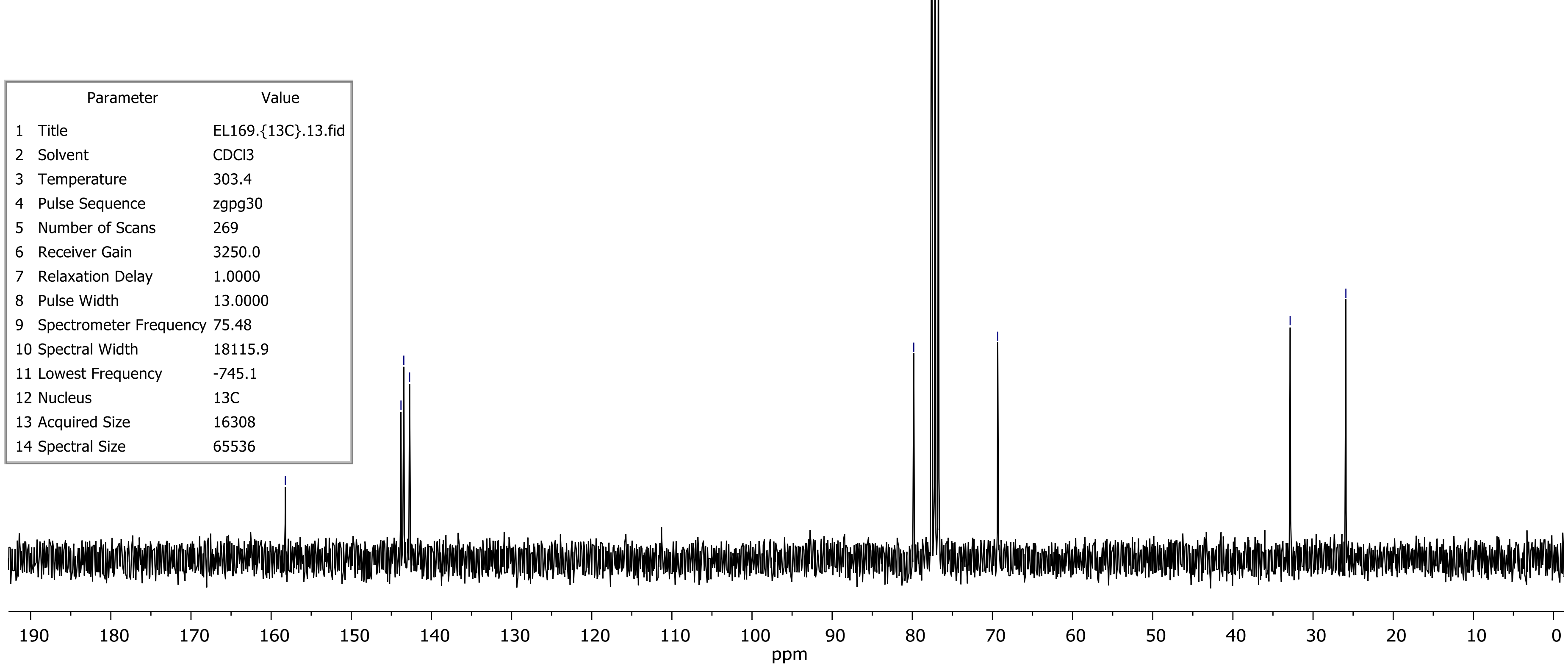

^{13}C NMR (75.48 MHz, $CDCl_3$)

2-(tetrahydrofuran-2-yl)pyrazine 3ga

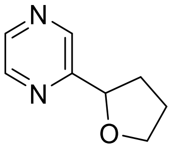

	Parameter	Value
1	Title	EL169.{13C}.13.fid
2	Solvent	CDCl3
3	Temperature	303.4
4	Pulse Sequence	zgpg30
5	Number of Scans	269
6	Receiver Gain	3250.0
7	Relaxation Delay	1.0000
8	Pulse Width	13.0000
9	Spectrometer Frequency	75.48
10	Spectral Width	18115.9
11	Lowest Frequency	-745.1
12	Nucleus	13C
13	Acquired Size	16308
14	Spectral Size	65536

	Parameter	Value
1	Title	EL599-1.{1H}.1.fid
2	Solvent	CDCl3
3	Temperature	298.0
4	Pulse Sequence	zg
5	Number of Scans	1
6	Receiver Gain	32.0
7	Relaxation Delay	0.1000
8	Pulse Width	14.7000
9	Spectrometer Frequency	300.23
10	Spectral Width	5882.4
11	Lowest Frequency	-1094.5
12	Nucleus	1H
13	Acquired Size	16308
14	Spectral Size	65536

^{1}H NMR (300.13 MHz, $CDCl_3$)

2-methyl-3-(tetrahydrofuran-2-yl)pyrazine 3ha

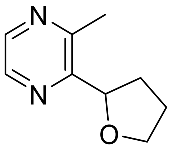

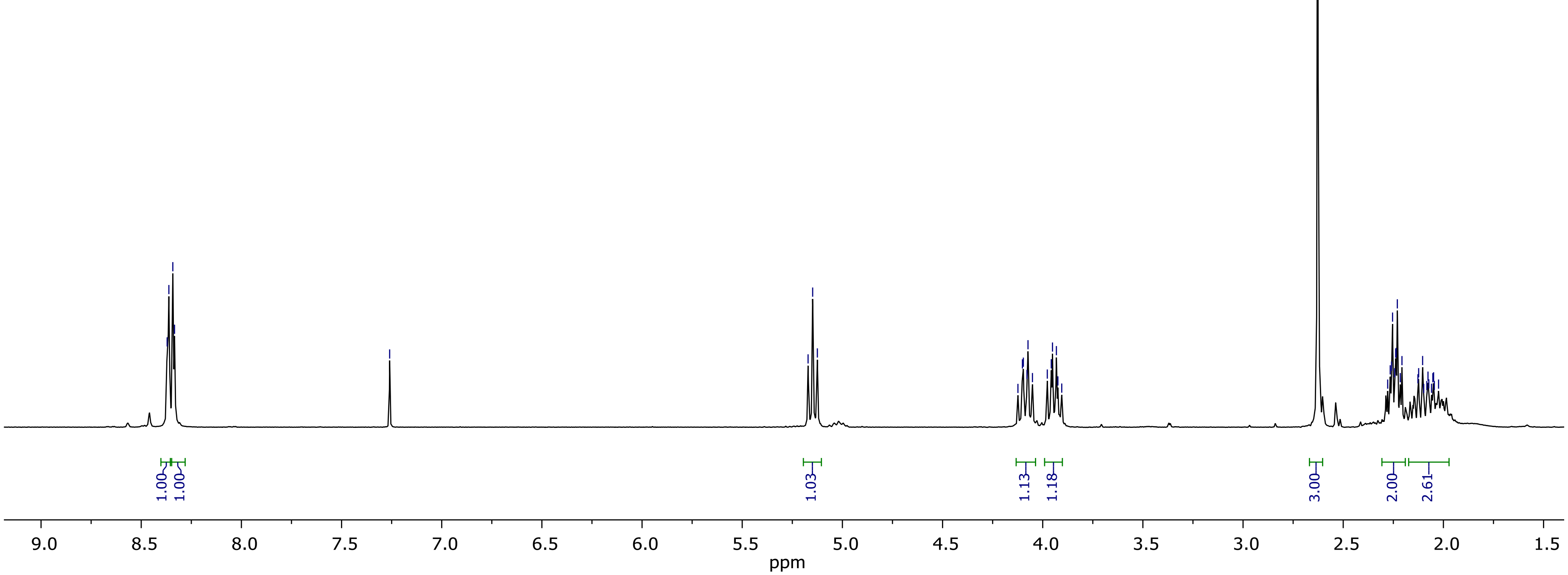

^{13}C NMR (75.48 MHz, $CDCl_3$)

2-methyl-3-(tetrahydrofuran-2-yl)pyrazine 3ha

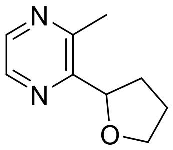

	Parameter	Value
1	Title	EL599-1.{13C}.2.fid
2	Solvent	CDCl3
3	Temperature	298.0
4	Pulse Sequence	zgpg30
5	Number of Scans	400
6	Receiver Gain	101.0
7	Relaxation Delay	0.8000
8	Pulse Width	9.9000
9	Spectrometer Frequency	75.50
10	Spectral Width	17857.1
11	Lowest Frequency	-1370.8
12	Nucleus	13C
13	Acquired Size	20412
14	Spectral Size	65536

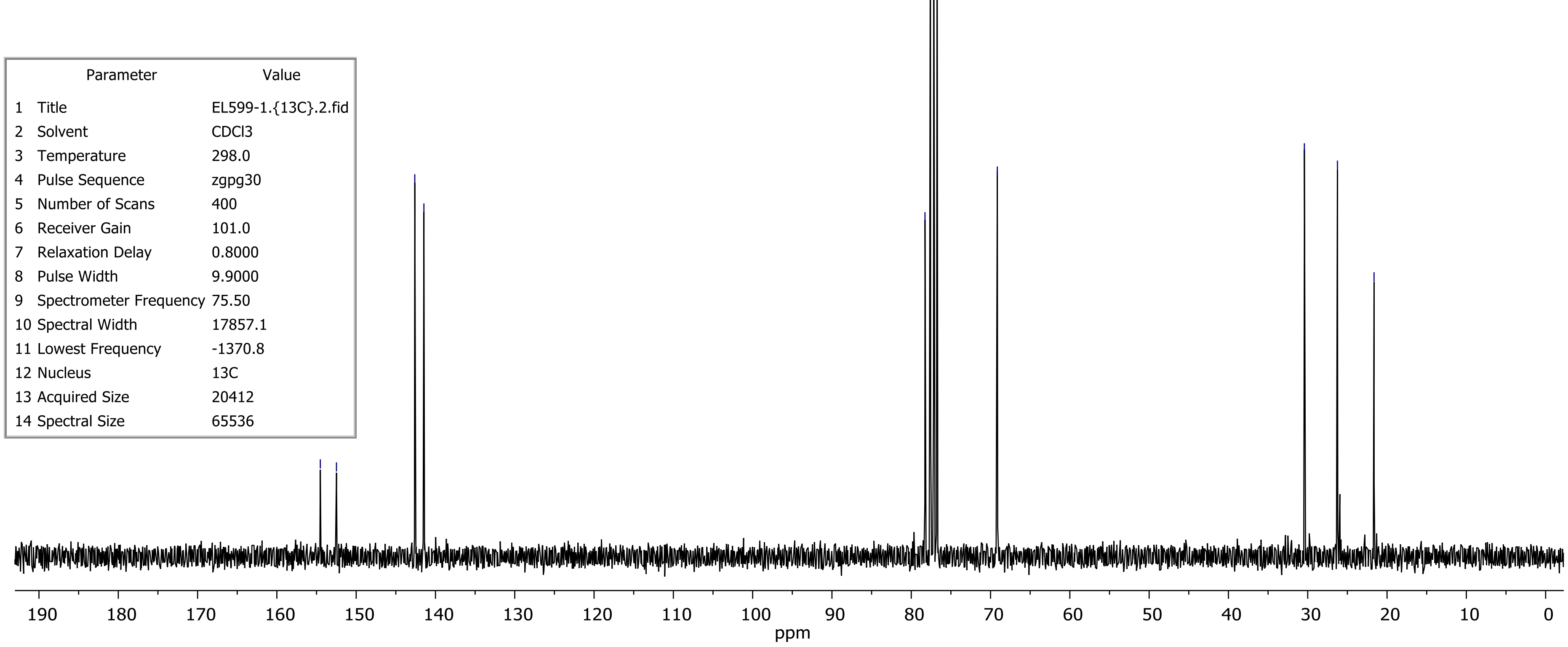

^{1}H NMR (300.13 MHz, $CDCl_3$)

methyl 6-(tetrahydrofuran-2-yl)nicotinate 3ia

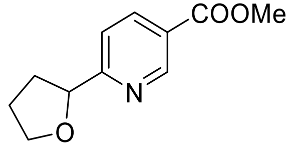

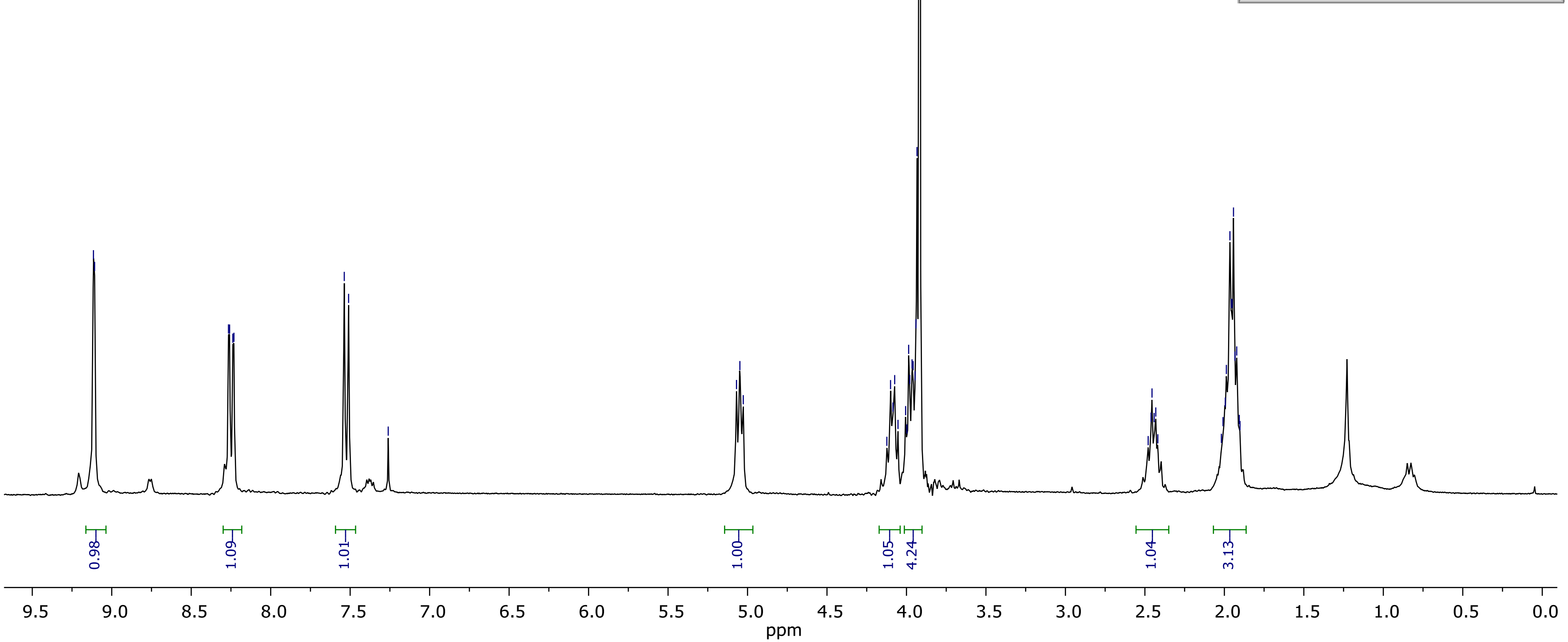

	Parameter	Value
1	Title	i7633.{1H}.1.fid
2	Solvent	CDCl3
3	Temperature	301.0
4	Pulse Sequence	zg
5	Number of Scans	1
6	Receiver Gain	28.5
7	Relaxation Delay	0.0050
8	Pulse Width	9.0000
9	Spectrometer Frequency	300.13
10	Spectral Width	6009.6
11	Lowest Frequency	-610.1
12	Nucleus	1H
13	Acquired Size	8124
14	Spectral Size	65536

i7633.{13C}.13.fid
/KANI NKA013a

^{13}C NMR (75.48 MHz, $CDCl_3$)

methyl 6-(tetrahydrofuran-2-yl)nicotinate 3ia

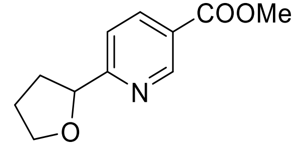

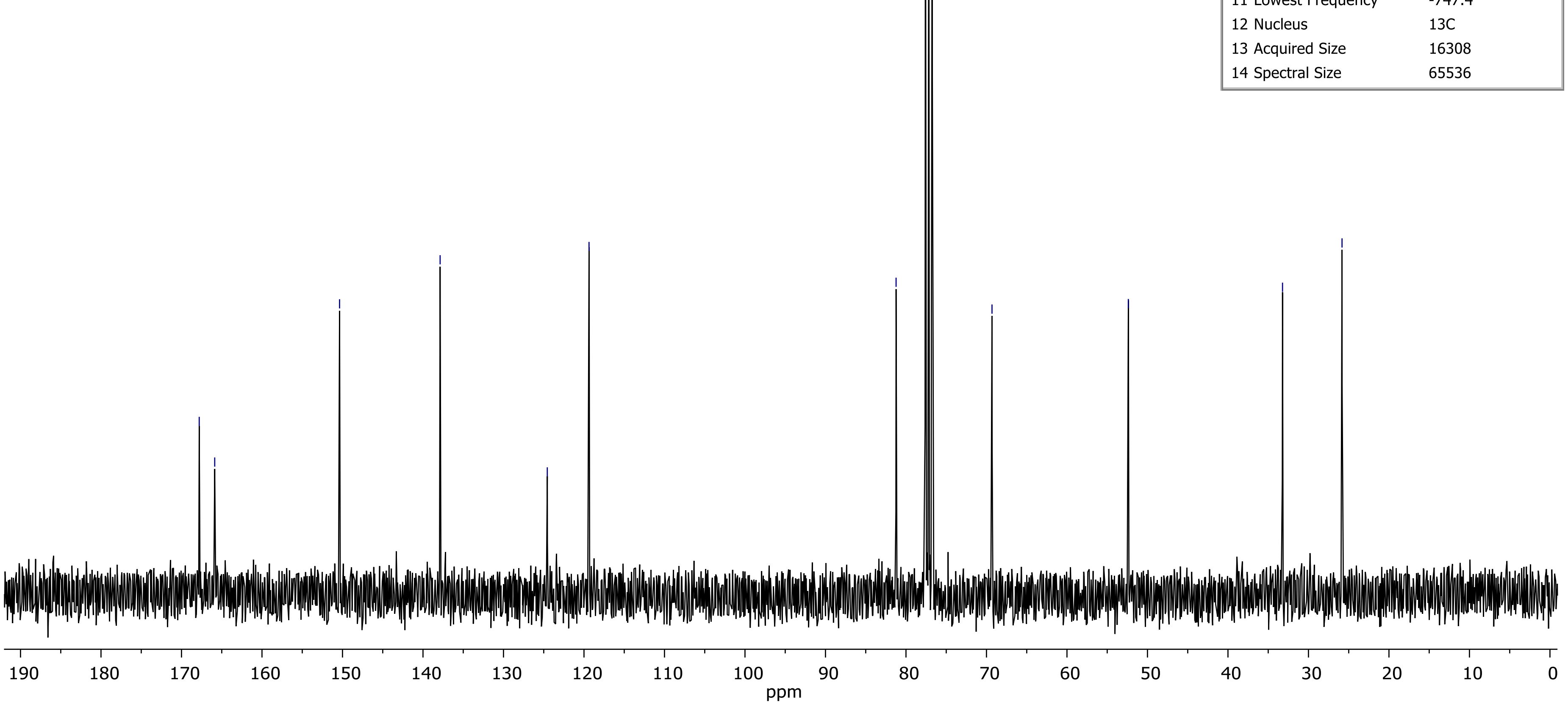

	Parameter	Value
1	Title	i7633.{13C}.13.fid
2	Solvent	CDCl3
3	Temperature	301.1
4	Pulse Sequence	zgpg30
5	Number of Scans	113
6	Receiver Gain	40.3
7	Relaxation Delay	1.0000
8	Pulse Width	13.0000
9	Spectrometer Frequency	75.48
10	Spectral Width	18115.9
11	Lowest Frequency	-747.4
12	Nucleus	13C
13	Acquired Size	16308
14	Spectral Size	65536

^{1}H NMR (300.13 MHz, $CDCl_3$)

4-methyl-2-(tetrahydrofuran-2-yl)quinoline 1-oxide 3ja

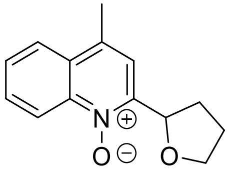

	Parameter	Value
1	Title	EL045.{1H}.1.fid
2	Solvent	CDCl3
3	Temperature	304.0
4	Pulse Sequence	zg
5	Number of Scans	1
6	Receiver Gain	40.3
7	Relaxation Delay	0.0050
8	Pulse Width	9.0000
9	Spectrometer Frequency	300.13
10	Spectral Width	6009.6
11	Lowest Frequency	-609.7
12	Nucleus	1H
13	Acquired Size	8124
14	Spectral Size	65536

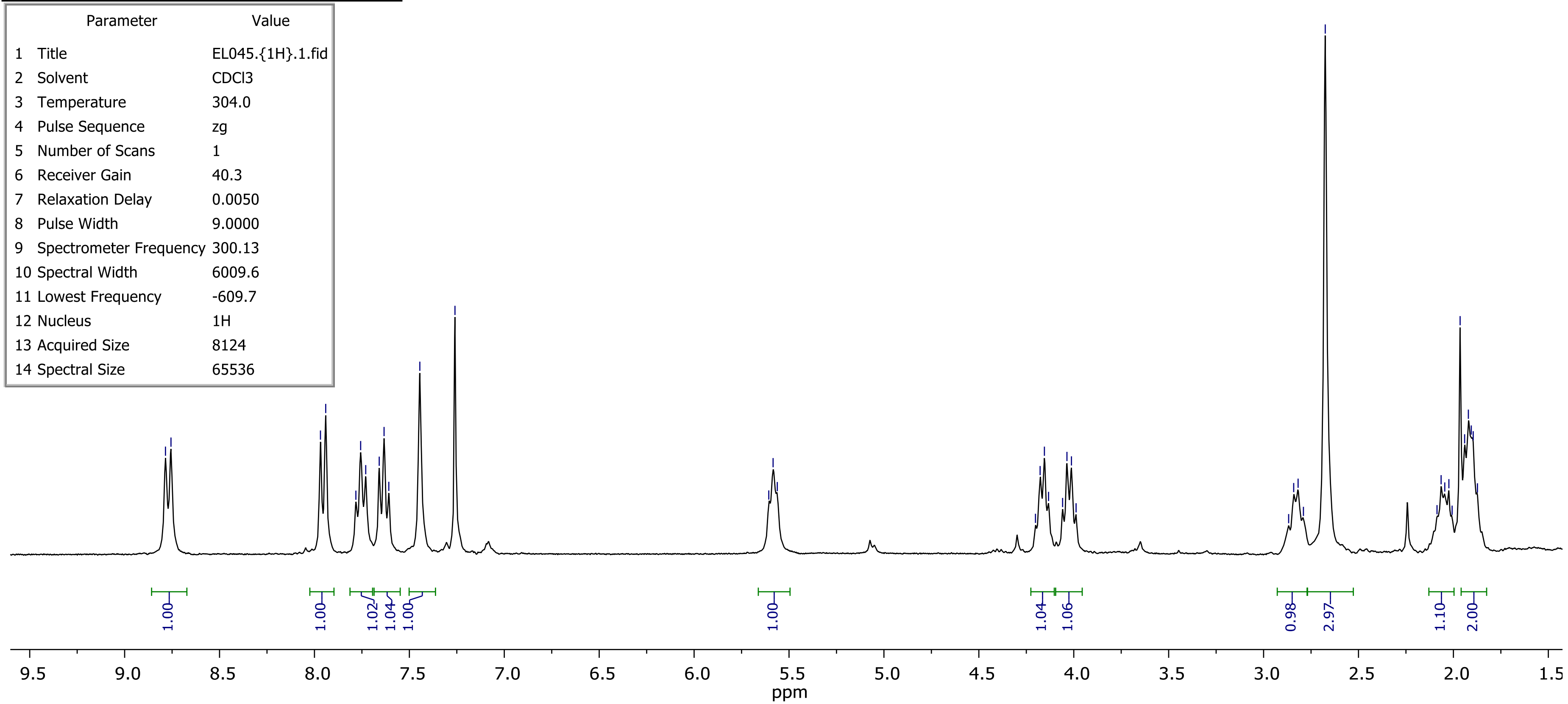

^{13}C NMR (75.48 MHz, $CDCl_3$)

4-methyl-2-(tetrahydrofuran-2-yl)quinoline 1-oxide 3ja

	Parameter	Value
1	Title	EL045.{13C}.13.fid
2	Solvent	CDCl3
3	Temperature	303.9
4	Pulse Sequence	zgpg30
5	Number of Scans	575
6	Receiver Gain	3250.0
7	Relaxation Delay	1.0000
8	Pulse Width	13.0000
9	Spectrometer Frequency	75.48
10	Spectral Width	18115.9
11	Lowest Frequency	-746.1
12	Nucleus	13C
13	Acquired Size	16308
14	Spectral Size	65536

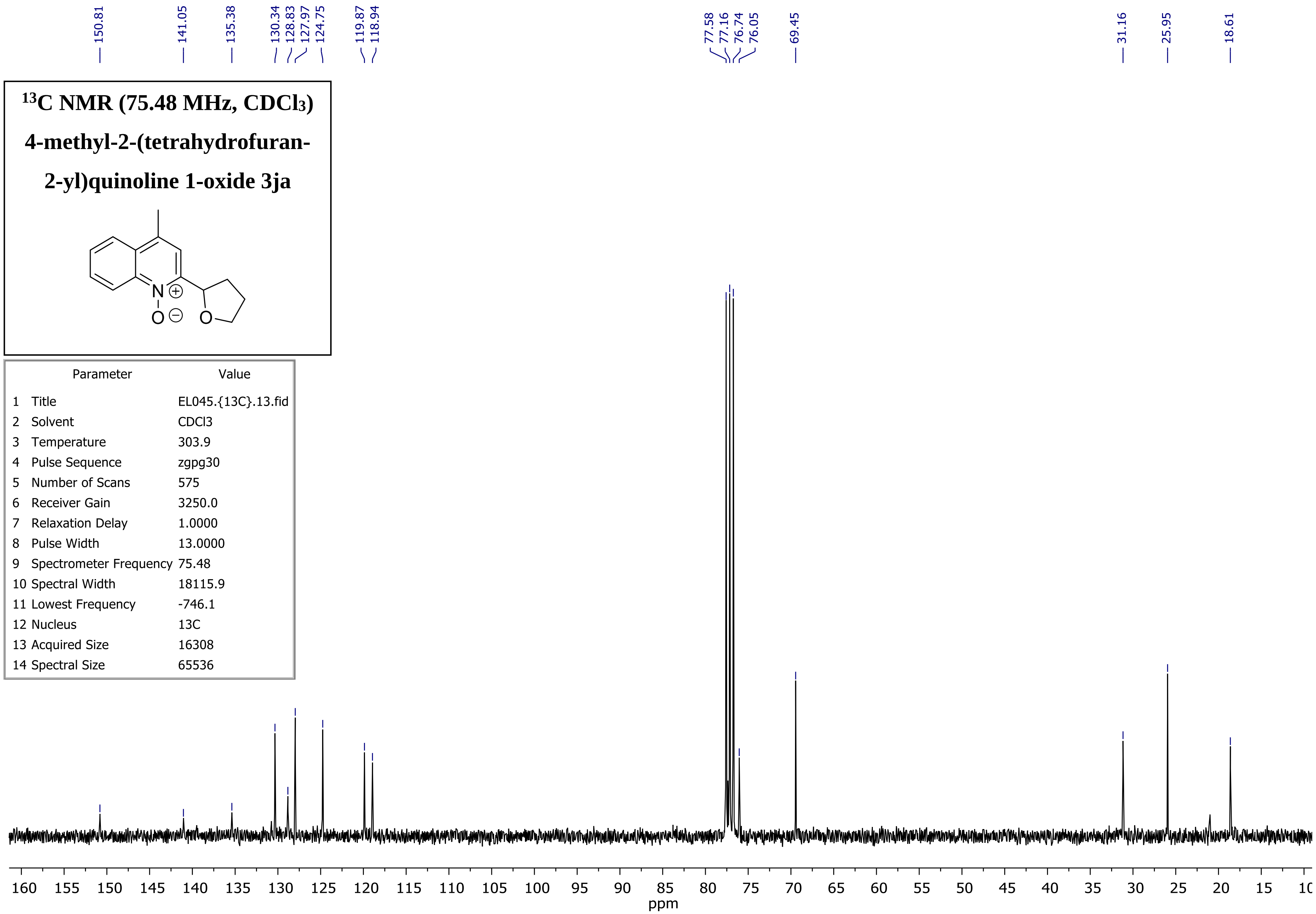

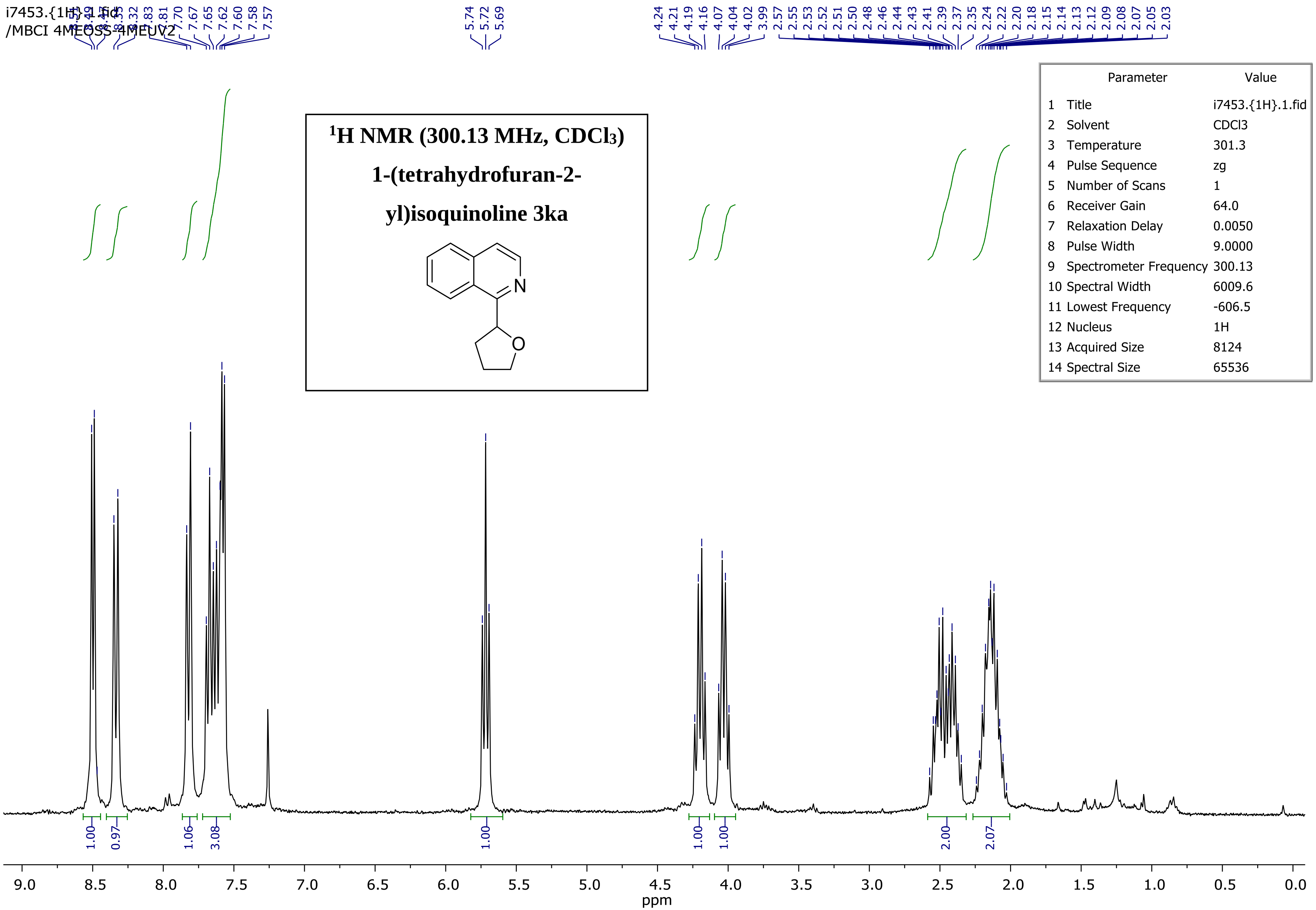

^{1}H NMR (300.13 MHz, $CDCl_3$)

1-(tetrahydrofuran-2-yl)isoquinoline 3ka

	Parameter	Value
1	Title	i7453.{1H}.1.fid
2	Solvent	CDCl3
3	Temperature	301.3
4	Pulse Sequence	zg
5	Number of Scans	1
6	Receiver Gain	64.0
7	Relaxation Delay	0.0050
8	Pulse Width	9.0000
9	Spectrometer Frequency	300.13
10	Spectral Width	6009.6
11	Lowest Frequency	-606.5
12	Nucleus	1H
13	Acquired Size	8124
14	Spectral Size	65536

i7535.{13C}.13.fid
/KANI NKA013a

^{13}C NMR (75.48 MHz, $CDCl_3$)

1-(tetrahydrofuran-2-yl)isoquinoline 3ka

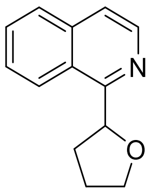

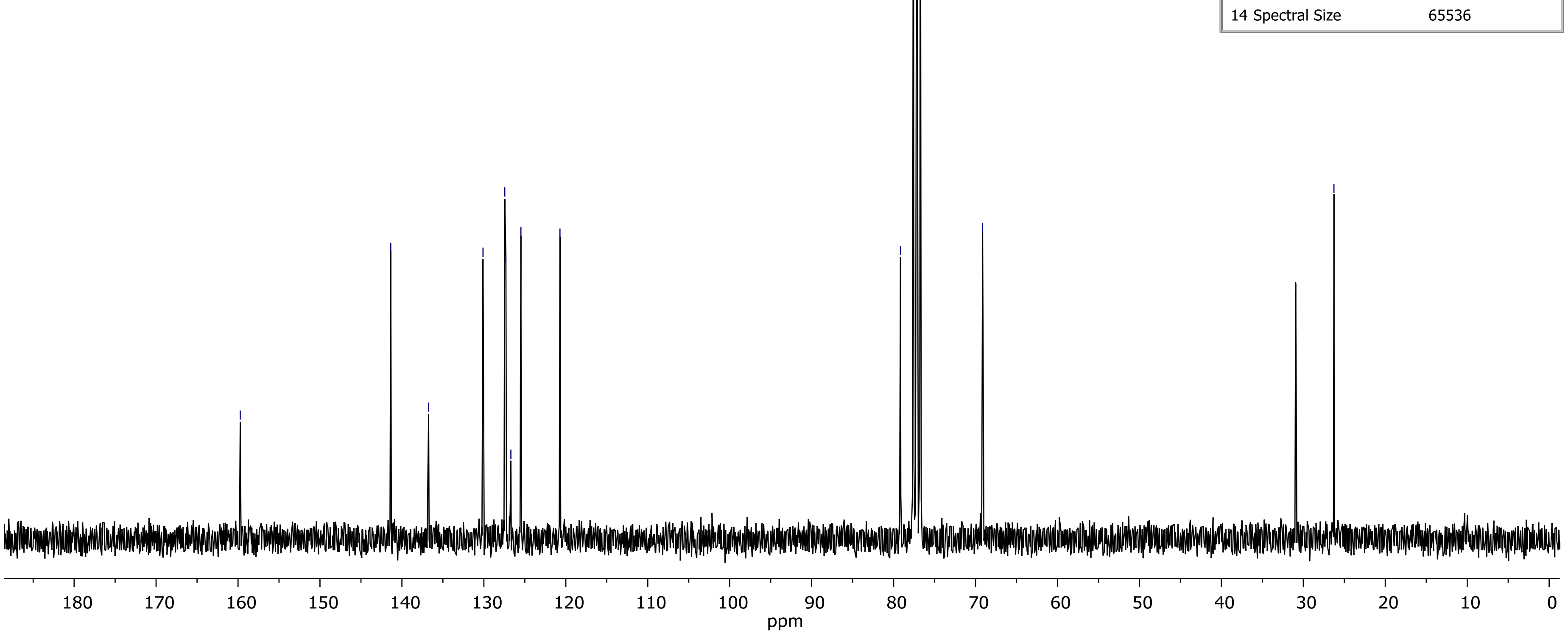

	Parameter	Value
1	Title	i7535.{13C}.13.fid
2	Solvent	CDCl3
3	Temperature	301.5
4	Pulse Sequence	zgpg30
5	Number of Scans	384
6	Receiver Gain	40.3
7	Relaxation Delay	1.0000
8	Pulse Width	13.0000
9	Spectrometer Frequency	75.48
10	Spectral Width	18115.9
11	Lowest Frequency	-746.8
12	Nucleus	13C
13	Acquired Size	16308
14	Spectral Size	65536

^{1}H NMR (300.13 MHz, $CDCl_3$)

2-(4-bromophenyl)imidazo[1,2-a]pyridine-3-carboxylic acid 3la’

	Parameter	Value
1	Title	i8738.{1H}.1.fid
2	Solvent	CDCl3
3	Temperature	301.4
4	Pulse Sequence	zg
5	Number of Scans	4
6	Receiver Gain	14.2
7	Relaxation Delay	0.0050
8	Pulse Width	9.0000
9	Acquisition Time	1.3518
10	Spectrometer Frequency	300.13
11	Spectral Width	6009.6
12	Lowest Frequency	-610.2
13	Nucleus	1H
14	Acquired Size	8124
15	Spectral Size	65536

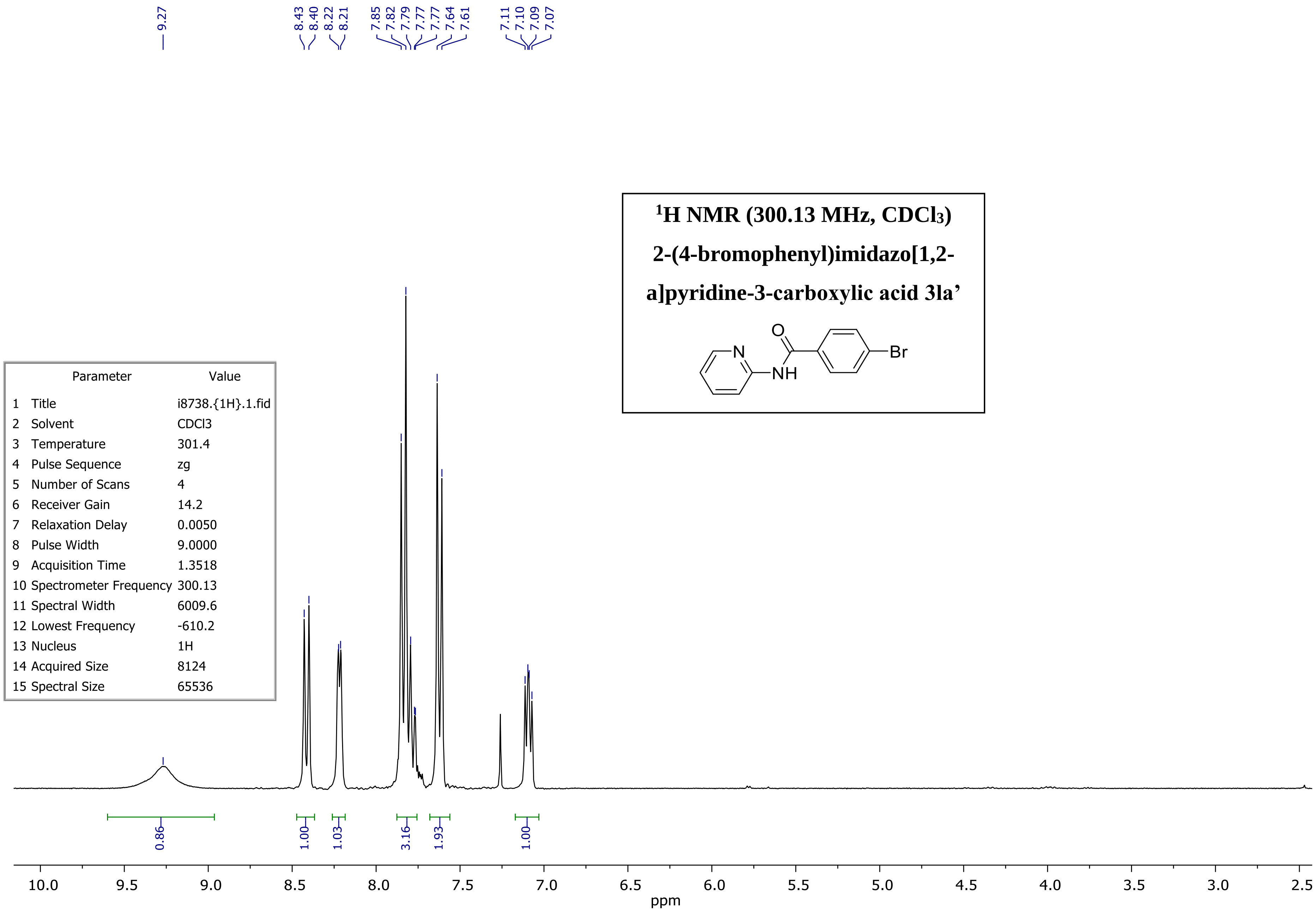

^{13}C NMR (75.48 MHz, $CDCl_3$)

2-(4-bromophenyl)imidazo[1,2-a]pyridine-3-carboxylic acid 3la’

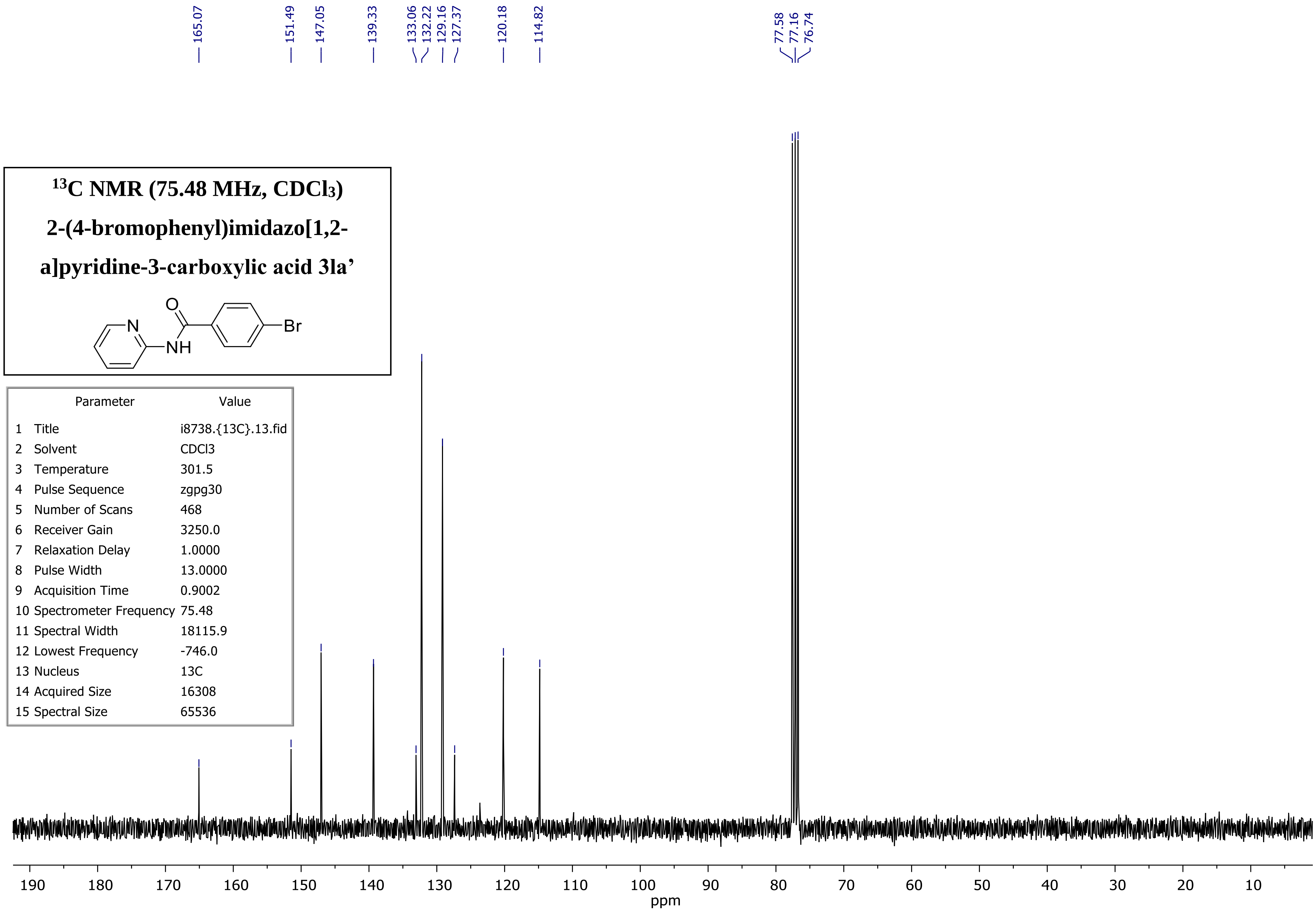

	Parameter	Value
1	Title	i8738.{13C}.13.fid
2	Solvent	CDCl3
3	Temperature	301.5
4	Pulse Sequence	zgpg30
5	Number of Scans	468
6	Receiver Gain	3250.0
7	Relaxation Delay	1.0000
8	Pulse Width	13.0000
9	Acquisition Time	0.9002
10	Spectrometer Frequency	75.48
11	Spectral Width	18115.9
12	Lowest Frequency	-746.0
13	Nucleus	13C
14	Acquired Size	16308
15	Spectral Size	65536
